# Multi-level functional genomics reveals molecular and cellular oncogenicity of patient-based 3′ untranslated region mutations

**DOI:** 10.1016/j.celrep.2023.112840

**Published:** 2023-07-28

**Authors:** Samantha L. Schuster, Sonali Arora, Cynthia L. Wladyka, Pushpa Itagi, Lukas Corey, Dave Young, Bethany L. Stackhouse, Lori Kollath, Qian V. Wu, Eva Corey, Lawrence D. True, Gavin Ha, Patrick J. Paddison, Andrew C. Hsieh

**Affiliations:** 1 Molecular and Cellular Biology Graduate Program, University of Washington, Seattle, WA 98195, USA; 2 Human Biology Division, Fred Hutchinson Cancer Center, Seattle, WA 98109, USA; 3 Public Health Sciences Division, Fred Hutchinson Cancer Center, Seattle, WA 98109, USA; 4 Department of Urology, University of Washington, Seattle, WA 98195, USA; 5 Clinical Research Division, Fred Hutchinson Cancer Center, Seattle, WA 98109, USA; 6 Department of Laboratory Medicine and Pathology, University of Washington, Seattle, WA 98195, USA; 7 Genome Sciences, University of Washington, Seattle, WA 98195, USA; 8 Department of Medicine, University of Washington, Seattle, WA 98195, USA; 9 Lead contact

## Abstract

3′ untranslated region (3′ UTR) somatic mutations represent a largely unexplored avenue of alternative oncogenic gene dysregulation. To determine the significance of 3′ UTR mutations in disease, we identify 3′ UTR somatic variants across 185 advanced prostate tumors, discovering 14,497 single-nucleotide mutations enriched in oncogenic pathways and 3′ UTR regulatory elements. By developing two complementary massively parallel reporter assays, we measure how thousands of patient-based mutations affect mRNA translation and stability and identify hundreds of functional variants that allow us to define determinants of mutation significance. We demonstrate the clinical relevance of these mutations, observing that CRISPR-Cas9 endogenous editing of distinct variants increases cellular stress resistance and that patients harboring oncogenic 3′ UTR mutations have a particularly poor prognosis. This work represents an expansive view of the extent to which disease-relevant 3′ UTR mutations affect mRNA stability, translation, and cancer progression, uncovering principles of regulatory functionality and potential therapeutic targets in previously unexplored regulatory regions.

## In brief

Using dual massively parallel reporter assays and CRISPR base editing, Schuster et al. demonstrate that 3′ untranslated region mutations found in advanced prostate cancer tumors significantly affect mRNA translation, mRNA stability, and cellular cancer phenotypes, uncovering alternative pathways of oncogenic dysregulation in 3′ UTR-mediated post-transcriptional gene expression.

## Graphical abstract

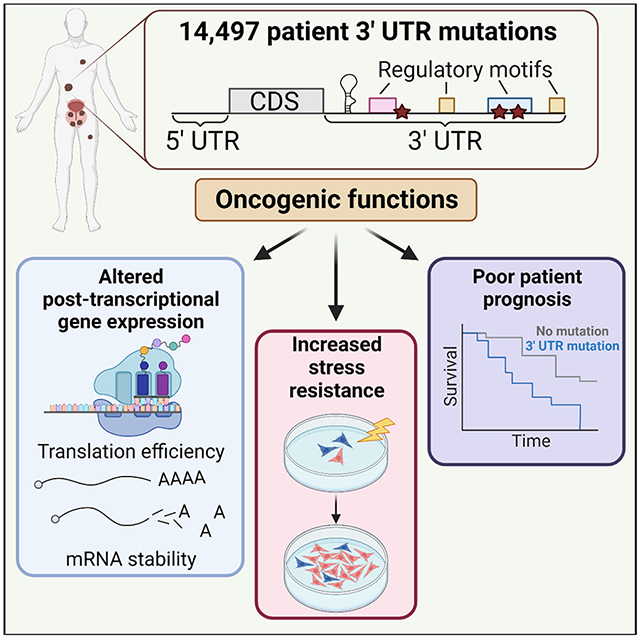

## INTRODUCTION

Metastatic, castration-resistant prostate cancer (mCRPC) is an advanced form of prostate cancer with a high mortality rate from a lack of curative treatment options.^1^ Although much is known about how coding sequence (CDS) mutations, mRNA expression changes, and genomic structural variations affect prostate cancer, these findings do not explain all aspects of disease pathogenesis, leaving significant gaps in our ability to treat patients.^2-7^ An alternative view of cancer gene regulation may give new insights as to how oncogenic pathways can be regulated in the absence of transcriptional changes. Importantly, only ~40% of protein expression can be explained by differences in mRNA expression, leaving much to be explored at the post-transcriptional level of oncogenic gene expression.^8^

3′ untranslated region (3′ UTR) somatic mutations represent a largely unexplored avenue for understanding aberrant gene regulation in cancer. The 3′ UTR of a gene is immediately downstream of the CDS and transcribed with it, but not translated. This region is a major mediator of mRNA stability and translation through sequence- and structure-based elements that recruit *trans*-acting factors. Many of these 3′ UTR-interacting factors, such as RNA-binding proteins (RBPs) and microRNAs (miRNAs), are dysregulated in cancer and contribute to pathogenesis.^9^ For example, binding of the RBP EBP1 to the androgen receptor (AR) 3′ UTR promotes its decay and translational repression and thereby acts as a tumor suppressor in prostate cancer.^10-12^ Additionally, the miRNA *miR-21* is oncogenic in prostate cancer, with overexpression increasing *in vitro* and *in vivo* growth.^13,14^ Although these *trans*-acting factors demonstrate that the 3′ UTR can be an important hub of cancer gene dysregulation, it is unknown how mutations to the 3′ UTR itself affect post-transcriptional dynamics in cancer.

Select studies have established the importance of individual 3′ UTR mutations in causing pathogenic changes in post-transcriptional gene regulation. For example, a 1–2 nt deletion in the *EGFR* 3′ UTR was found to increase *EGFR* mRNA stability in colorectal tumors, thereby increasing cell sensitivity to EGFR inhibition.^15^ Another study found a T→C single-nucleotide variant (SNV), also in the *EGFR* 3′ UTR, that abolished a *miR-103a-3p* binding site, thereby increasing *EGFR* expression and derepressing cell growth.^16^ These studies demonstrate how 3′ UTR mutations can lead to actionable overexpression patterns similar to more well-known amplifications or protein-coding mutations; however, such functional 3′ UTR mutation studies are currently limited to individual variants. Massively parallel reporter assays (MPRAs) are powerful tools that combine reporter plasmid libraries with next-generation sequencing, enabling systematic analysis of gene expression changes caused by thousands of sequences of interest simultaneously.^17^ They have recently been used to study basic principles of 3′ UTR-mediated gene regulation, using random or evolutionarily conserved sequences to identify patterns of function within this region.^18-23^ However, the impact patient-based 3′ UTR mutations have on post-transcriptional gene expression has not been explored in this high-throughput manner.

In this study, we have combined 3′ UTR somatic mutation calling of 185 mCRPC patient tumors with dual MPRA technology to investigate how thousands of 3′ UTR single-nucleotide mutations affect mRNA translation and stability. We find hundreds of patient-derived mutations that significantly change post-transcriptional gene regulation and determine specific sequence features associated with mutation function. CRISPR-Cas9 base editing allows us to further explore patient mutations within their endogenous genomic contexts, demonstrating that single-nucleotide changes in *ZWILCH* and *IGF1R* 3′ UTRs are sufficient to increase protein expression and cellular growth. We further explore patient outcome data, finding that oncogenic 3′ UTR mutations that change mRNA translation or stability are associated with faster progression to advanced disease. This comprehensive study establishes the functional significance and clinical relevance of patient-based 3′ UTR mutations, demonstrating their ability to drive molecular and cellular oncogenicity.

## RESULTS

### mCRPC patients harbor thousands of 3′ UTR mutations in cancer-related genes and regulatory elements

**Figure 1.**
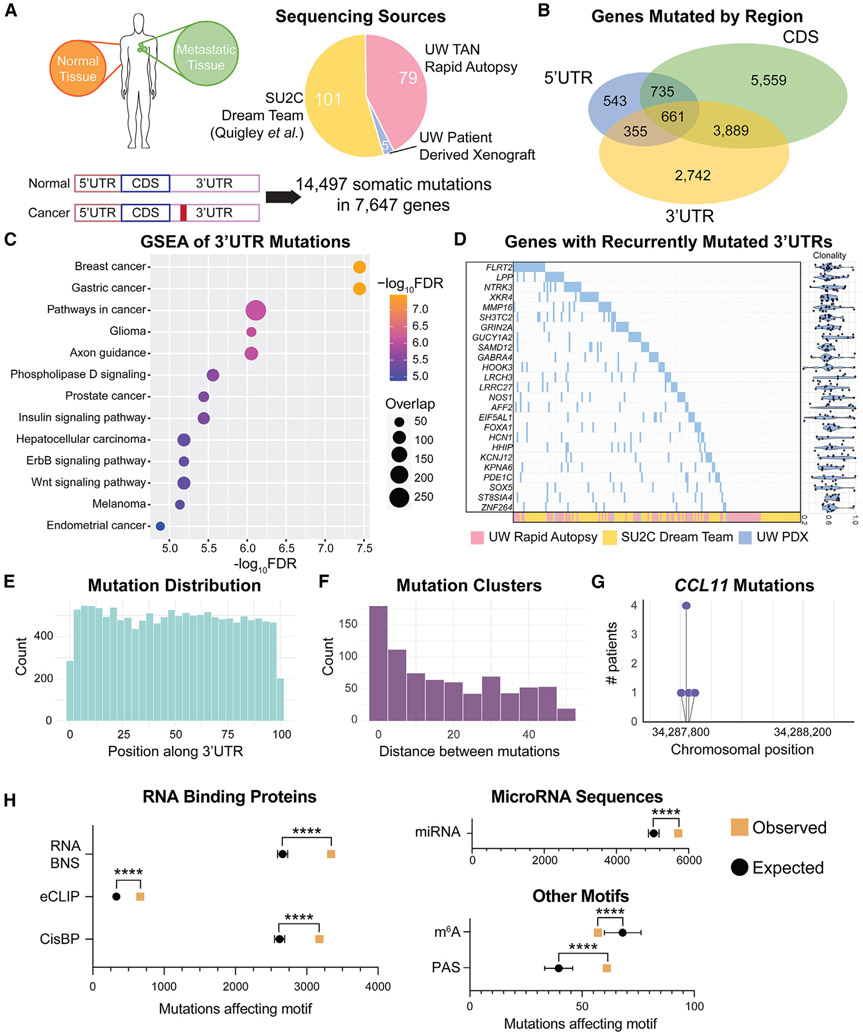
mCRPC patients harbor thousands of 3′ UTR mutations in cancer-related genes and regulatory elements (A) Schematic of patient sequencing sources and mutation calling process. (B) Overlap between genes mutated in 5′ UTR, CDS, and/or 3′ UTRs. (C) KEGG_2021_Human gene set enrichment analysis (GSEA) for genes containing 3′ UTR mutations in mCRPC patients. (D) Genes with top recurrently mutated 3′ UTRs, with x axis indicating patients containing a 3′ UTR in each gene. Per patient clonality (percent of tumor cells bearing a 3′ UTR mutation in the given gene) is shown to the right. (E) Metaplot of mutation position along the 3′ UTR for all 14,497 mutations, normalized for each gene’s 3′ UTR length. (F) Histogram of distances between all 3′ UTR mutations found within 50 bases of another patient mutation. (G) Lollipop plot showing patient mutations in *CCL11* 3′ UTR. (H) *In silico* analysis of observed versus expected number of mutations affecting 3′ UTR regulatory elements. Statistical analysis conducted using one sample t tests (mean ± SD, ****p < 0.0001). RNA Bind-N-Seq (RNA BNS), ENCODE RNA eCLIP, Catalog of inferred sequence Binding Preferences of RBPs (CisBP).

To study how 3′ UTR mutations affect post-transcriptional gene expression in prostate cancer, we first built a UTR-centric sequencing database including whole-genome sequencing (WGS) or UTR sequencing of 185 mCRPC tumors. This dataset consists of in-house UTR-specific sequencing of matched normal and metastatic tissue for 79 patients from the University of Washington (UW) Tissue Acquisition Necropsy (TAN) program^24,25^ and 5 samples from the UW LuCaP patient-derived xenograft (PDX) models.^17^ Additionally, we obtained WGS of matched normal and metastatic tissue for 101 mCRPC patients from the Stand Up to Cancer (SU2C) West Coast Dream Team project^3^ (Figure 1A). In addition to UTR sequencing, exome sequencing was available for 47 of the UW patients, enabling us to compare the 3′ UTR mutational landscape to that of the 5′ UTR and CDS across 148 of our patients. To call high-confidence somatic mutations, we implemented a combination of four mutation callers (Mutect2,^26^ Strelka2,^27^ MuSE,^28^ and Varscan2^29^) over both the UTRs and protein-coding space, using read cutoffs according to the sequencing depth of each dataset (Figure S1A; Table S1). For all further analysis, we retained only mutations that had been found in at least two callers (Figure S1B), amounting to 14,497 3′ UTR somatic single-nucleotide mutations across 7,647 genes. About two-thirds (67.6%) of these mutations were identified by all four callers, demonstrating a high level of agreement. Furthermore, 95% of the 3′ UTR mutations called by our pipeline in the SU2C cohort were also called in the original published SU2C study, confirming that our pipeline has called a reliable set of 3′ UTR mutations in these patient tumors (Figure S1C).

Importantly, we observe minimal bias in our ability to call mutations across these different patient cohorts. The ratio of UW to SU2C patients containing 3′ UTR mutations in each gene averages 1.05. The few instances where this ratio is skewed may be explained by regional differences in sequence coverage (*FLRT2, XKR4, LPP*, and *ST8SIA4*; Figure S1D) or biological differences among the cohorts (*EIF5AL1* and *GUCY1A2*), wherein the UW tumor samples represent a more advanced stage of disease than SU2C tumors.

To further establish the robustness of these 3′ UTR mutations, we compared the tumor mutational burden (TMB) and mutational signatures between the UTR and CDS regions in these patients. We find the overall TMB of the 3′ UTR to be slightly lower than that of the CDS in mCRPC patients, but equivalent to the 5′ UTR and within the range of previously published prostate cancer TMB^30^ (Figure S1E). The 3′ UTR mutations largely follow expected patterns of base substitutions, similar to that of the CDS, displaying a strong bias toward C→T/G→A transition mutations (Figure S1F). To expand on such base change patterns, the COSMIC project has defined mutational signatures across cancer types in terms of base substitutions and their surrounding trinucleotide context.^31^ In our dataset, both 3′ UTR and CDS mutations follow established mutational signature patterns, with most patients exhibiting COSMIC signature 1, known to be found in almost all cancers, and many following signatures 5 and 6, which are specifically associated with prostate cancer (Figure S1G). Additionally, we find enrichment of signatures 3, 8, and 16 specifically in 3′ UTR mutations. Signature 3 is associated with failure to repair DNA double-stranded breaks, while signatures 8 and 16 have unknown etiology. The differences in mutational signatures between the CDS and 3′ UTR potentially demonstrate variable mutational constraints and selective pressures upon these genomic regions. Overall, the 3′ UTR mutations called in our dataset follow expected patterns for prostate cancer, while uncovering alterations unique to this region.

These 3′ UTR mutations greatly expand the space of potentially oncogenic mutations in mCRPC tumors, as many 3′ UTR mutations fall into genes without CDS mutations, but with known relevance to cancer (Figures 1B and 1C). In fact, we observe significant enrichment of 3′ UTR mutations in cancer-related gene sets and pathways, including breast, gastric, and prostate cancer signatures, suggesting that these mutations may be an important source of aberrant oncogenic expression (Figure 1C). We identify several well-studied cancer-associated genes with highly mutated 3′ UTRs. The highest burden of 3′ UTR mutations are found in *FLRT2* and *LPP* (mutated in 20 and 15 patients, respectively), which are both cell adhesion proteins associated with cancer (Figure 1D). *LPP* is known to have roles in regulating both metastasis and treatment resistance^32-35^ and *FLRT2* has been identified as tumor suppressive in several cancers.^36-38^ We also find that established regulators of prostate cancer *FOXA1* and *SOX5* each contain 3′ UTR mutations across 9 different patient tumors (4.8%).^39-42^ Interestingly, some of these 3′ UTR mutations are clonal in the patient tumors, while the average mutation is present in ~60% of tumor cells (Figures 1D and S1H). The enrichment of 3′ UTR mutations in established cancer-related genes leads us to hypothesize that these mutations are an alternative method of oncogenic dysregulation.

In addition to analyzing the genes targeted by 3′ UTR mutations, we endeavored to delineate patterns of mutation across individual 3′ UTR sequences. We observe that mutations are found equally distributed along the length of the 3′ UTR, not enriched near the CDS nor the poly-A tail (Figure 1E). However, we find more than 700 loci where multiple 3′ UTR mutations are found within 50 bases of each other, with more than 250 of these being within 5 bases of one another (Figure 1F). Formal significance analysis using FishHook^43^ finds two 3′ UTRs (*CCL11* and *ZNF492*) recurrently mutated more than expected across our cohort. In particular, *CCL11*, a chemokine implicated in lung and ovarian cancer, contains four 3′ UTR mutations clustered within 12 nt across 7 patients (Figure 1G).^44,45^ These clustered mutations suggest repeated targeting of functional sequence motifs within the 3′ UTR. Therefore, we set out to determine whether each 3′ UTR mutation in our dataset could change known RBP motifs, miRNA seed sequences, the poly-A signal (PAS) AAUAAA,^46^ or the m^6^A RRACH motif.^47^ We then compared these results with an *in silico* analysis of 14,497 mutations randomly distributed across the 3′ UTRome, keeping base changes and trinucleotide context of the mutations constant. We observe that patient 3′ UTR mutations alter RBP, miRNA, and PAS motifs significantly more than expected by chance, while they are excluded from m^6^A motifs (Figure 1H). Given the prevalence of mutations in cancer genes and regulatory motifs, we hypothesize that these mutations may be functional in altering 3′ UTR-mediated gene expression, and therefore significant in cancer. This analysis has defined the landscape of 3′ UTR mutations in advanced prostate cancer tumors, uncovering a wealth of potentially interesting mutations in cancer-related genes and *cis*-regulatory elements, and now functional assays are necessary to determine which of these mutations are driver alterations.

### Patient-based 3′ UTR mutations functionally affect gene-specific translation efficiency

**Figure 2.**
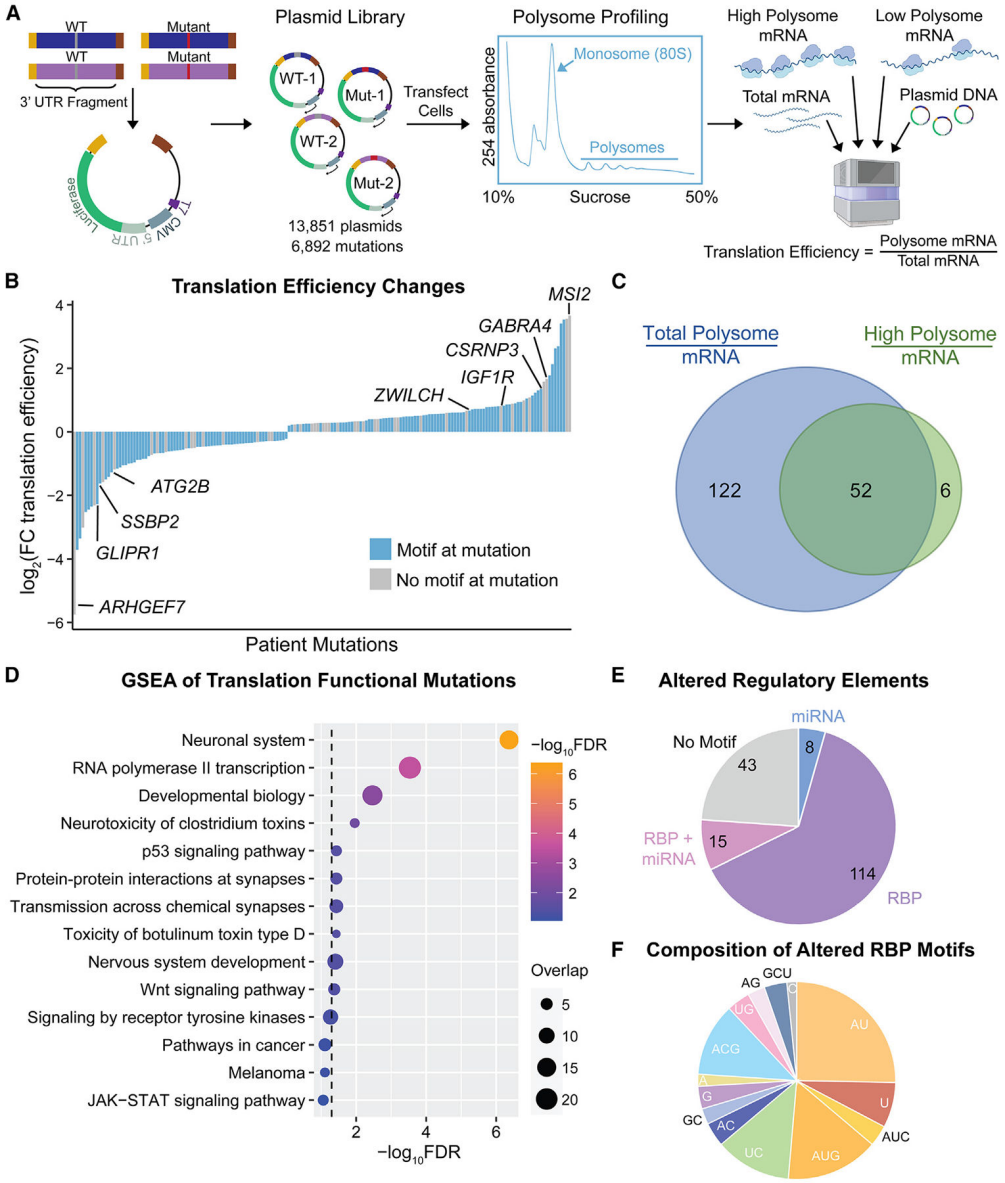
Patient-based 3′ UTR mutations functionally affect gene-specific translation efficiency (A) Schematic showing MPRA plasmid library construction, polysome profiling, and sequencing of distinct RNA and DNA samples. (B) All 3′ UTR mutations significantly changing translation efficiency as determined by total polysome/total mRNA ratio (n = 6). Mutations in known regulatory elements indicated in blue. (C) Overlap between 3′ UTR mutations significantly changing TE according to total-polysome/mRNA ratio versus high-polysome/mRNA ratio. (D) Kyoto Encyclopedia of Genes and Genomes (KEGG) and Reactome (MSigDB) gene set enrichment analysis for the genes containing 3′ UTR mutations that significantly affect TE. Dotted line, FDR = 0.05. (E) Proportion of functional 3′ UTR mutations that alter miRNA, RBP, and/or no motif. (F) Base compositions of RBP motifs altered by functional 3′ UTR mutations.

To functionally assess how patient mutations change 3′ UTR-mediated aspects of post-transcriptional gene regulation, we first built a polysome profiling-based MPRA able to simultaneously measure how thousands of mutations change translation efficiency (TE) (Figure 2A). Two hundred base pair sequences of the 3′ UTR around each of 6,892 mutations from recurrently mutated genes were inserted downstream of the luciferase CDS in a modified pGL4 plasmid backbone, pLuc2CP-noARE (Table S2). Sequencing of this plasmid library confirmed adequate and even representation of 3′ UTR inserts (Figure S2A).

The plasmid library was transfected into PC3 cells, chosen to represent the mCRPC cellular environment similar to that of patient samples. Polysome profiling was performed on the transfected cells to fractionate mRNA by the number of attached ribosomes, resulting in distinct monosome-, low polysome-, and high polysome-associated pools of mRNA. Six biological replicates of the polysome-associated mRNA pools, plus total mRNA and plasmid DNA extracted from each sample, were sequenced. TE was calculated on the basis of the ratio of total polysome- or high polysome-associated mRNA to total mRNA for each 3′ UTR insert, and wild-type and mutant pairs of 3′ UTR inserts were analyzed for significant TE changes (false discovery rate [FDR] < 0.10) caused by each mutation using xtail (Table S3).

We achieved an average sequencing depth of 315 reads per insert in each sample, even sequencing across replicates and sample types, and high correlation between biological replicates in each sample group (Figures S2B-S2D). Internal control sequences from published 3′ UTR MPRAs were included to validate our methods. First, we found several “activator” or “repressor” sequences from Oikonomou et al.^20^ that significantly (FDR < 0.10) changed TE relative to the average TE across all MPRA 3′ UTR inserts, and 4/5 of these behave as originally published (Figure S2E). Also, addition of the Pumilio binding element shows expected results agreeing with the PTRE-seq MPRA, causing a dose-dependent decrease in RNA expression while TE remains stable^19^ (Figure S2F). These quality control checks indicate robustness across our assay.

We discover 180 3′ UTR point mutations that significantly change TE, with a more than 1,000-fold dynamic range (Figure 2B). As expected, the two methods of calculating TE, either using total or high polysome-associated mRNA, agree well, with total polysomes uncovering a larger pool of functional mutations (Figure 2C). To ensure that these MPRA observations are robust, we validated several top hits using individual dual luciferase assays, observing expected changes in protein expression in accordance with those observed by polysome profiling (Figures 2B and S2G). We also confirmed that these protein expression changes were translation dependent by normalizing dual luciferase assay results to mRNA expression levels (Figure S2H). Furthermore, our MPRA results correlate well with *in vivo* TE changes observed via ribosome profiling, a method that measures TE similar to polysome profiling, of tumor samples from the five UW PDX models. With these data, we correlate tissue-level TE changes for a 3′ UTR-mutated gene with the behavior of the same mutation in our MPRA, finding that 5 of 8 mutation effects observed by both MPRA and ribosome profiling agree, with a Spearman correlation coefficient of r = 0.55 (Figure S2I). These experiments show the power of single-nucleotide 3′ UTR mutations to affect translation dynamics on a large scale and demonstrate the applicability of our high-throughput reporter technology to *in vivo* tissue effects.

Many functional 3′ UTR mutations cause seemingly oncogenic changes in expression, either increasing TE of oncogenic mRNAs or decreasing TE of tumor suppressive genes (Figure 2B). For example, we find a particular 3′ UTR mutation in *MSI2* (chr17:57681293 A→G) that increases its TE almost 16-fold. We would expect this change to be impactful in a tumor setting, as *MSI2* is an RBP previously shown to promote progression in prostate and other cancer types.^48-50^ Additionally, *GLIPR1* and *SSBP2* have tumor suppressive qualities in multiple cancers, including prostate, making their suppressive 3′ UTR mutations (chr12:75503808 T→C, 4.8-fold decrease, and chr5:81416226 G→A, 3.1-fold decrease, respectively) potentially clinically relevant.^51-56^ Interestingly, we find enrichment of functional mutations in neuronal genes, suggesting dysregulation of neuroendocrine-related pathways that are often a means of cellular plasticity and therapy resistance in advanced prostate cancer^57^ (Figure 2D). There is also significant enrichment across cancer-related gene sets, including p53, Wnt, receptor tyrosine kinase, and JAK-STAT signaling, highlighting the many oncogenic pathways dysregulated by 3′ UTR mutations.

To determine how these 3′ UTR mutations affect TE on a molecular level, we analyzed whether they alter known regulatory sequences, finding that more than 75% of functional mutations change RBP and/or miRNA binding motifs (Figure 2E). Specifically, most mutations are in RBP binding sites, particularly those that bind AU- and U-rich motifs, including hnRNPCL1, PUF60, RBM6, and RC3H1^58^ (Figure 2F). Intriguingly, we also find 43 mutations that affect TE in the absence of a clear *cis*-regulatory element, suggesting the involvement of RNA structural changes or novel motifs. This MPRA uncovers the effects patient-derived 3′ UTR mutations have on mRNA translation, establishing the broad importance of these mutations in regulating oncogenic expression through altering sequence elements.

### Patient-based 3′ UTR mutations significantly alter oncogenic mRNA stability

**Figure 3.**
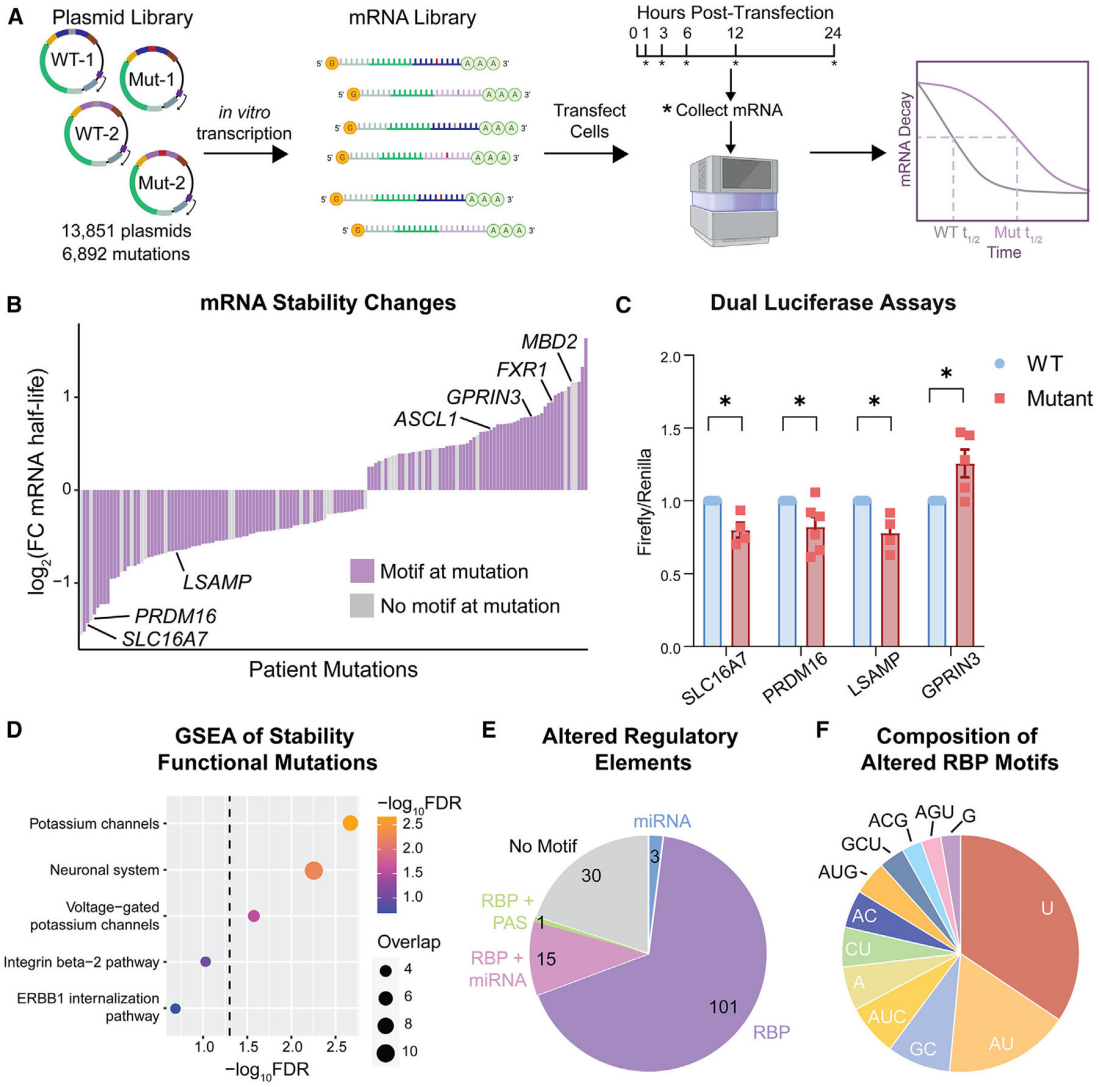
Patient-based 3′ UTR mutations significantly alter oncogenic mRNA stability (A) Schematic of mRNA stability MPRA. Plasmid library is *in vitro* transcribed to an mRNA library, which is transfected into cells, and a 24 h RNA-seq time course is conducted to measure changes in mRNA decay. (B) Fold changes in mRNA half-life of all 3′ UTR mutations causing significant changes in mRNA stability (n = 6). Mutations in known regulatory elements indicated in purple. (C) Results of dual luciferase assays performed on four functional 3′ UTR mutations. Statistical analysis conducted using unpaired t test and multiple comparisons correction of Benjamini, Krieger, and Yekutieli (*q < 0.05, mean ± SEM, n = 4, 6, 4, and 5). (D) Reactome 2019 gene set enrichment analysis for the genes containing 3′ UTR mutations that significantly affect mRNA stability. Dotted line, FDR = 0.05. (E) Proportion of functional 3′ UTR mutations that alter miRNA seed sequence, RBP motif, polyadenylation signal (PAS), and/or no motif. (F) Base compositions of RBP motifs altered by functional 3′ UTR mutations.

In addition to TE, the 3′ UTR is an important mediator of mRNA stability. To determine how mCRPC patient mutations affect mRNA degradation, we designed and implemented a second, complementary MPRA using an RNA sequencing (RNA-seq) time course of *in vitro* transcribed (IVT) mRNA (Figure 3A). We used the integrated T7 promoter to *in vitro* transcribe our plasmid library into a fully capped and poly-A tailed mRNA library. We transfected PC3 cells with this mRNA library, then collected library mRNA from the cells over a 24 h time course in 6 biological replicates. An exogenous spike-in was used to normalize between time points and replicates, ensuring quantitative measurement of mRNA degradation. The RNA-seq time course was sequenced to an average depth of 443 reads per insert, with uniform depth and high correlation across samples and replicates (Figures S3A-S3C).

To define the decay of each mRNA from the 3′ UTR library, we fit non-linear least-squares regression curves with the formula $y_{t}=y_{plateau}+(y_{0}+y_{plateau})*e^{-\alpha (t)}$ to the data for each 3′ UTR insert and calculated mRNA half-lives using the relationship $halflife=\frac{\text{ln}\;(2)}{\alpha }$ (Figure S3D). As most of the dynamic range between different 3′ UTRs occurs at the 3 and 6 h time points, these were used for statistical analysis, in addition to filtering by raw read counts and changes in mRNA half-life (Table S4). Using these criteria, we observe expected results in several internal control 3′ UTRs. The insertion of miRNA seed sequences decreases mRNA stability relative to a blank control and mutating the seed sequences abrogates this repression (Figure S3E). Furthermore, the addition of RBP motifs, including an AU-rich element (ARE) and a Pumilio element, both cause expected decreases in mRNA stability^19,59^ (Figure S3F). We also externally validated several MPRA hits using separate dual luciferase reporter assays, finding expected alterations in protein expression (Figures 3B and 3C). We further confirm that the 3′ UTR mutation in *SLC16A7* works specifically through decreasing mRNA stability using actinomycin D transcriptional shutoff (Figure S3G). Additionally, we find that changes in mRNA stability measured by our MPRA generally agree with tissue-level mRNA expression changes in UW PDX tumor samples (Figure S3H; 6/8 mutation agreement).

Overall, we find 150 patient-based 3′ UTR mutations that significantly change mRNA stability, many of which are in known oncogenes (Figure 3B). Tumor mutations in *ASCL1*, a driver of advanced neuroendocrine prostate cancer,^60^ *MBD2*, an epigenetic regulator that binds methylated DNA,^61,62^ and the RBP *FXR1*^63,64^ each increase the half-lives of their respective oncogenic mRNAs, establishing these as functional oncogenic 3′ UTR mutations (Figure 3B, chr18:54154901 T→A, chr12:102960501 T→A, and chr3:180978239 A→G, respectively).

Collectively, the functional mutations affecting mRNA stability are enriched in neuronal genes (Figure 3D). Together with our previous findings (Figure 2D), this strengthens the hypothesis that 3′ UTR mutations can be an alternative way neuroendocrine features are dysregulated in advanced prostate cancer. To investigate the extent to which 3′ UTR mutation function is cell state specific, we performed dual luciferase assays in the hormone sensitive prostate adenocarcinoma LNCaP cell line to compare with our findings from the neuroendocrine-like, castration-resistant PC3 cell line (Figure S3I). We find both cell-type-dependent and independent mutation effects, with three 3′ UTR mutations in *ARHGEF7, SLC16A7*, and *LSAMP* functioning the same in both cell lines. Interestingly, the 3′ UTR mutation in the GAB_A_ neurotransmitter receptor subunit *GABRA4* is the only mutation to have opposite effects in the two cell lines, substantiating the idea that there may be differences in 3′ UTR-mediated regulation of distinct neuronal genes in advanced prostate cancer cells.

Like those mutations that affect TE, most of these stability-affecting 3′ UTR mutations alter known RBP binding sequences, particularly those with AU- and U-rich motifs, pointing to the dual effects many RBPs have on both mRNA translation and stability (Figures 3E and 3F). Our complementary MPRA technologies allow us to measure how 3′ UTR mutations affect two distinct stages of post-transcriptional gene regulation and observe the common theme that functional mutations on either level target established cancer-related genes and ablate RBP binding elements.

### 3′ UTR mutation functionality is determined by sequence conservation, regulatory motifs, and RNA structure

**Figure 4.**
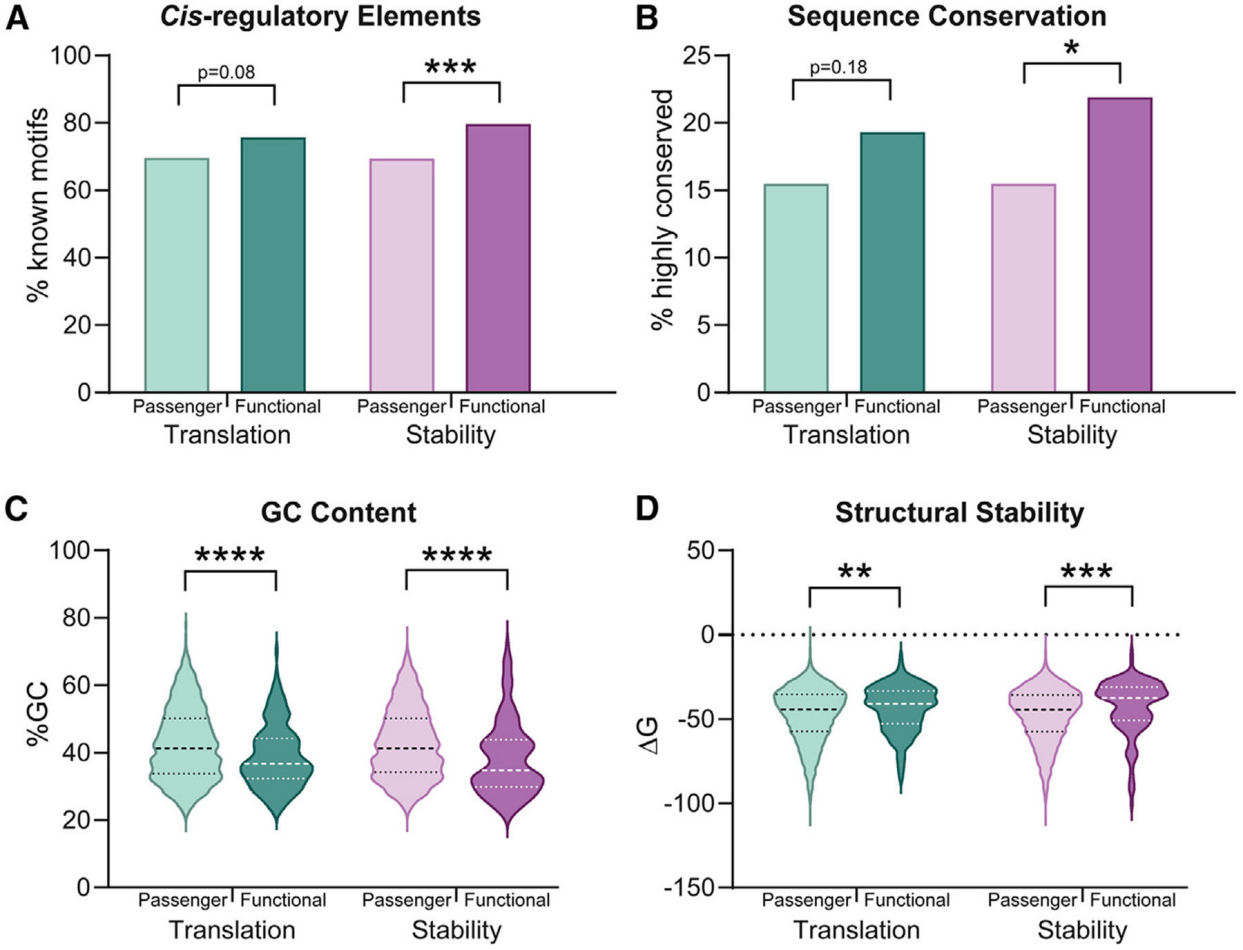
3′ UTR mutation functionality is determined by sequence conservation, regulatory motifs, and RNA structure (A and B) Percentage of passenger or functional 3′ UTR mutations tested by translation or stability MPRAs that alter known regulatory motifs (A) or are highly evolutionarily conserved (PHASTCONS score > 0.9) (B). Statistical analysis conducted using a chi-square test (*p < 0.05 and ***p < 0.001). (C and D) GC content (C) and ΔG of RNAfold predicted secondary structure (more negative indicates more stable structure; D) for each passenger or functional 3′ UTR mutation. Statistical analysis conducted using two-tailed unpaired t tests (**p < 0.01, ***p < 0.001, and ****p < 0.0001).

Using the power of our dual MPRAs, we have tested thousands of patient-derived 3′ UTR mutations and identified hundreds that change gene expression, allowing us to delineate aspects of 3′ UTRs connected to mutation function. What distinguishes passenger from functional 3′ UTR mutations is a critical question for understanding 3′ UTR biology and how it can contribute to dysregulation in disease. We have noted that most functional 3′ UTR mutations alter known *cis*-regulatory elements. Comparing this with the background level of passenger mutations that fall in regulatory elements without changing gene expression, we find that stability-modifying mutations alter motifs significantly more than passenger mutations (Figure 4A). As regulatory motifs are often under selective pressure to remain functional throughout evolution, it follows that functional mutations may target conserved sequences. As expected, we observe that stability-related functional mutations are also found significantly more in areas of high sequence conservation (Figure 4B, Table S5). These trends toward functional mutations preferentially being in conserved, *cis*-regulatory element sequences also holds true for 3′ UTR mutations altering TE, though they are less pronounced. A possible explanation may be that translation-mediating 3′ UTR mutations are less dependent on sequence elements because they can act through structural changes instead. In fact, we see that 3′ UTR mutations that change either mRNA translation or stability have significantly lower GC content and less structural stability (less negative ΔG of the predicted minimum free energy secondary structure) than passenger mutations, suggesting that areas of lower RNA structural stability are more likely to be functional when mutated (Figures 4C and 4D). From this analysis, we have uncovered important determinants of 3′ UTR mutation function, whereby mutations in highly conserved regulatory motifs are more likely to affect mRNA stability, and those in areas of low structure are likely to change multiple levels of 3′ UTR-mediated post-transcriptional gene regulation.

### Patient-based 3′ UTR mutations affect post-transcriptional gene expression and cancerous phenotypes in endogenous cellular contexts

**Figure 5.**
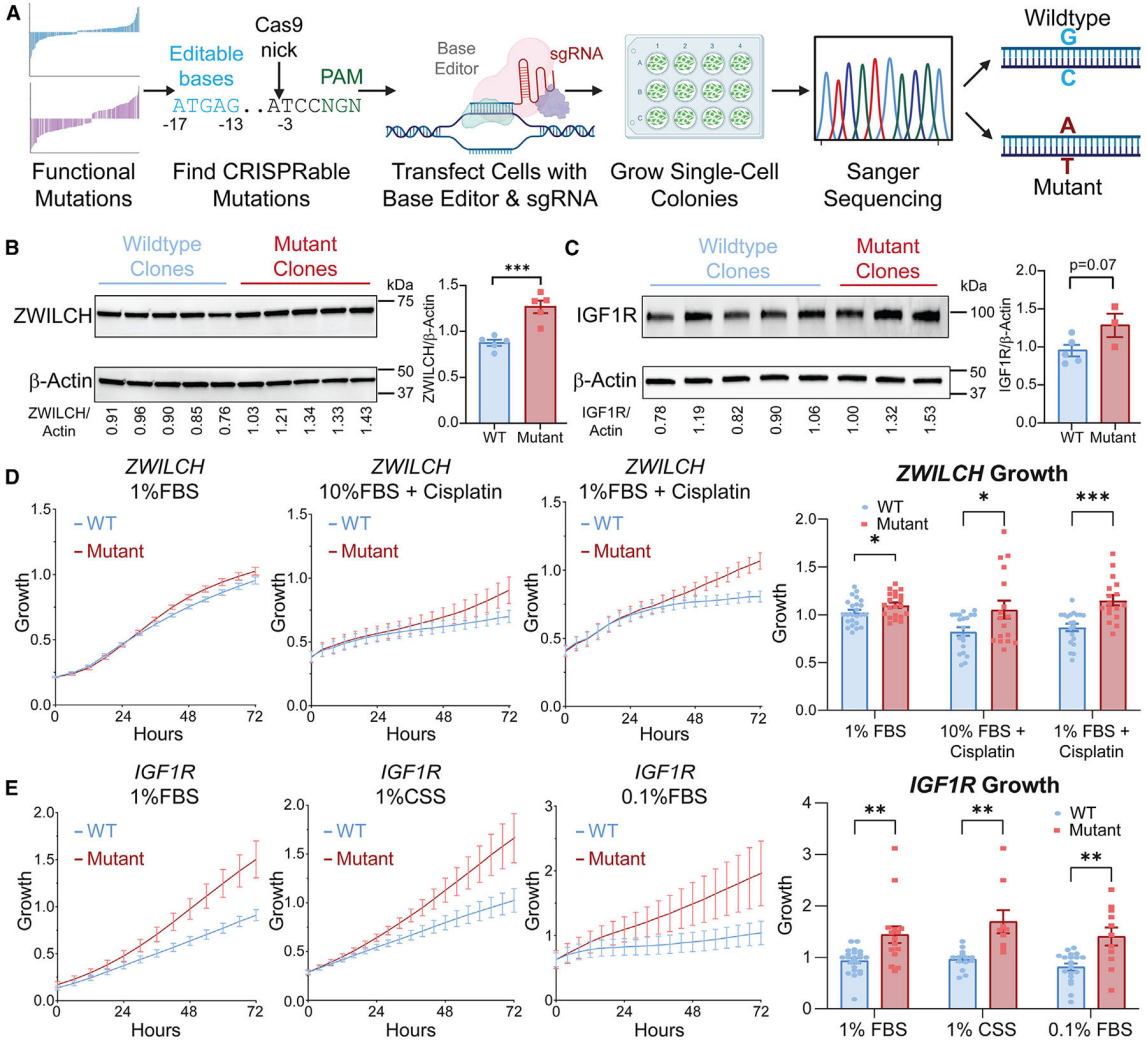
Patient-based 3′ UTR mutations affect post-transcriptional gene expression and cancerous phenotypes in endogenous cellular contexts (A) MPRA hits were analyzed to determine whether they could be CRISPR base edited, then cells were transfected with corresponding base editors and sgRNAs to grow out single-cell clonal wild-type and 3′ mutant cell lines confirmed by Sanger sequencing. (B and C) Western blot of endogenous ZWILCH (B) or IGF1R (C) and β-actin for individual wild-type and 3′ mutant clonal cell lines. ImageJ-calculated ratios of ZWILCH or IGF1R to β-actin protein levels shown below blots and summarized at right (mean ± SEM). Statistical analysis conducted using two-tailed unpaired t tests (***p < 0.001). ZWILCH, n = 5 WT and 5 mutant lines; IGF1R, n = 5 and 3. (D and E) Left: growth of *ZWILCH* (D) and *IGF1R* (E) wild-type and 3′ mutant cell lines under various stress conditions (mean ± SEM). Right: endpoint growth at 72 h using unpaired t test and multiple comparisons correction of Benjamini, Krieger, and Yekutieli (mean ± SEM; *p < 0.05, **p < 0.01, and *** p <0.001). ZWILCH, n = 24/21, 20/17, and 20/17 biological replicates (WT/mutant lines); IGF1R, n = 25/12, 20/12, and 15/9.

Our systems-based analysis has expanded our understanding of 3′ UTR mutation biology; however, it remains to be determined how these mutations impact cellular function. To address this, we used CRISPR-Cas9 base editing to explore the cellular consequences of 3′ UTR mutations in cancer. CRISPR base editors allow us to introduce 3′ UTR mutations precisely into their endogenous genomic location; however, there are specific constraints on what mutations can be created on the basis of PAM availability, potential off-target effects, and the desired base change.^65^ Therefore, we computationally sorted through the top potentially oncogenic 3′ UTR mutations from our M PRAs (those that increase gene expression of oncogenic or pro-proliferative mRNAs) to determine which could be mutated using available CRISPR base editing systems, resulting in the choice of two translation-enhancing mutations in *ZWILCH* and *IGF1R* (chr15:66548998 A→G and chr15:98958058 G→A) (Figure 5A). We transfected cells with CRISPR base editing systems specific to these mutations and selected single-cell clones, resulting in the development of several wild-type and mutant clonal cell lines for each mutation confirmed by Sanger sequencing (Figures S4A and S4B).

Western blotting for the endogenous ZWILCH and IGF1R proteins confirms that these single-nucleotide 3′ UTR mutations increase protein expression (~45% in ZWILCH, ~35% in IGF1R; Figures 5B and 5C). These changes in overall protein levels are especially striking when considering that the 3′ UTR mutation was only introduced to one genomic allele (the *ZWILCH* locus being diploid and *IGF1R* locus tetraploid in these cells). We observe no change in mRNA expression in the 3′ mutant cell lines, validating our MPRA results where these mutations specifically changed TE (Figures S4C and S4D). We next sought to determine whether the increased protein expression conferred by these 3′ UTR mutations had cellular, potentially oncogenic, effects.

ZWILCH is an essential component of the mitotic spindle assembly checkpoint necessary for pausing metaphase transition to anaphase until chromosomes are properly attached to the kinetochore.^66^ Overexpression of *ZWILCH* and other related mitotic checkpoint proteins has been previously associated with cancer progression,^67-69^ and there is a strong negative correlation between *ZWILCH* mRNA levels and patient survival in the SU2C dataset^6^ (Figure S4E). As such, we sought to determine how this 3′ UTR mutation affects cell growth. Although there is no difference between *ZWILCH* wild-type and 3′ UTR mutant cell lines in fully supplemented media (Figure S4F), mutant lines grow significantly faster when under stress conditions. In particular, we challenged the cells with either nutrient deprivation or cisplatin, finding that the 3′ UTR mutation confers a growth advantage in each condition (Figure 5D). The observed cisplatin resistance is especially striking, as this DNA-damaging agent is a direct challenge to the mitotic checkpoint and has clinical implications for chemotherapy use.

IGF1R is a growth factor receptor involved in the progression of multiple cancer types through its activation of several downstream oncogenic pathways.^70-73^ While under basal conditions, this *IGF1R* mutation does not change cell growth rates (Figure S4G), when cells’ access to IGF1R ligands was reduced by either decreasing FBS or replacing it with charcoal-stripped serum,^74^ we observe a growth advantage in the mutant cells (Figure 5E). This suggests that 3′ UTR-mediated *IGF1R* overexpression enables cells to activate proliferative signaling in response to minimal growth factor. These findings demonstrate that 3′ UTR mutations can increase cancer cell oncogenicity by enhancing growth under stress, a quality particularly important within the harsh ecosystem of a tumor microenvironment.

### Molecular mechanisms of 3′ UTR mutations in *ZWILCH* and *IGF1R*

**Figure 6.**
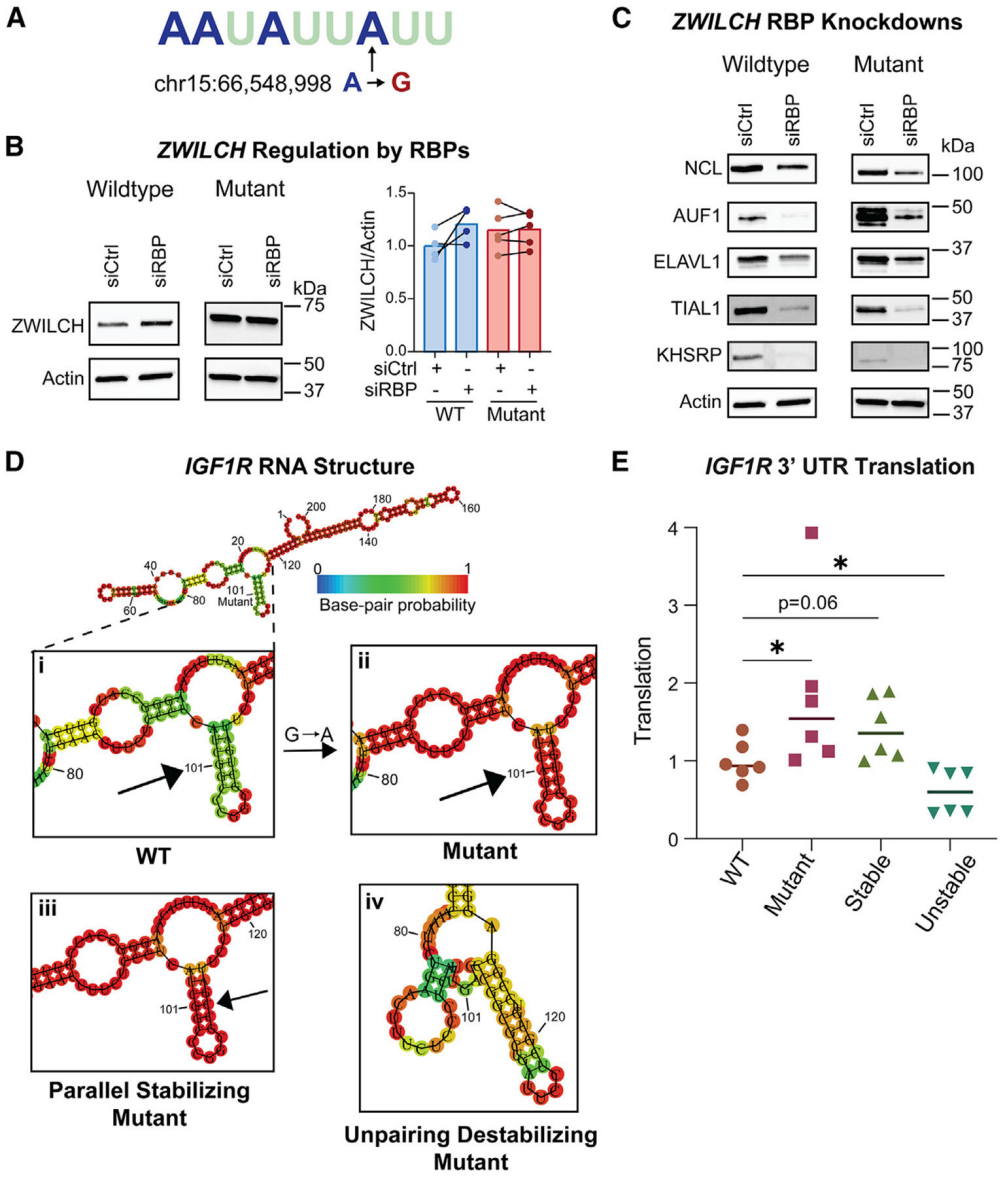
Molecular mechanisms of 3′ UTR mutations in *ZWILCH* and *IGF1R* (A) Sequence context around *ZWILCH* 3′ UTR mutation. (B) Change in ZWILCH protein expression by western blot upon simultaneous knockdown of NCL, AUF1, ELAVL1, TIAL1, and KHSRP (siRBP) in wild-type versus 3′ UTR mutant cells. One representative western blot shown at left, quantification of all replicates shown at right (n = 4 wild-type, n = 5 mutant). (C) Representative western blots showing simultaneous knockdown of NCL, AUF1, ELAVL1, TIAL1, and KHSRP in wild-type and 3′ UTR mutant cells. (D) RNAfold predicted secondary structure of WT *IGF1R* 3′ UTR sequence (200 bases around 3′ UTR mutation) shown at top. Below are shown changes in the predicted structure around (ii) the original G→A patient mutation, (iii) an independent stabilizing T→C mutation, and (iv) a destabilizing CGG→TGA mutation, where the altered mutations of interest are denoted by arrows. (E) Changes in translation from *IGF1R* 3′ UTR mutations shown in D, quantified by dual luciferase assays normalized to luciferase mRNA qPCR ([Firefly RLU/Renilla RLU]/Firefly mRNA). Statistical analysis conducted using Mann-Whitney tests (*p < 0.05, n = 6; lines denote medians).

Next, we turned to elucidating the mechanisms by which these *ZWILCH* and *IGF1R* mutations improve translation. Examining the sequence around each mutation for predicted changes in *cis*-regulatory elements, we find that the *ZWILCH* 3′ UTR mutation sits within an ARE, altering an adenine to a guanine (Figure 6A). AREs are common repressive elements of the 3′ UTR that can be bound by many different RBPs. To determine what RBPs are involved in the regulation of *ZWILCH* translation through this ARE, we analyzed a group of five candidate ARE-binding RBPs that are known to repress translation and highly expressed in PC3 cells: NCL, AUF1, ELAVL1, TIAL1, and KHSRP.^75-79^ Although knocking down each of these AU-binding RBPs individually does not change ZWILCH expression, we find that knockdown of all five RBPs together increases ZWILCH protein expression specifically in wild-type cells (Figures 6B and 6C). This indicates that multiple RBPs can bind this ARE and compensate for each other when only one is knocked down. Importantly, this ARE-RBP regulation of translation is negated by mutation of the *ZWILCH* ARE, where cells containing the A→G 3′ UTR mutation do not display increased ZWILCH expression upon multi-RBP knockdown. Therefore, we conclude that multifactorial repression of wild-type *ZWILCH* translation occurs through a combination of ARE-binding RBPs, where an A→G 3′ UTR mutation can disrupt ARE-RBP binding to derepress translation.

Interestingly, the mutation in *IGF1R* falls within the minority of functional mutations that do not alter a known regulatory motif. However, upon probing of the predicted RNA secondary structure using RNA-fold,^80,81^ we find that this mutation causes a considerable change in the stability of a particular stem-loop structure. The G→A mutation introduces a proper A-U Watson-Crick base pair to the stem in place of a G-U pair, increasing the likelihood of this stem-loop structure being formed (Figures 6Di and 6Dii). We therefore set out to determine the relationship between RNA structure and *IGF1R* translation by engineering other mutations that alter the stem-loop’s stability. First, we introduced a parallel T→C mutation 10 bases downstream of the original mutation of interest, stabilizing the stem-loop by creating a proper G-C pair in place of the original G-U pair (Figure 6Diii). Excitingly, we find that this mutation increases translation from the *IGF1R* 3′ UTR similarly to our original mutation of interest (Figure 6E). Furthermore, we disrupted the stem-loop’s stability by unpairing two nucleotides surrounding the original mutation of interest (CGG→TGA at bases 100–102, Figure 6Div). This mutation causes a significant decrease in translation from the *IGF1R* 3′ UTR (Figure 6E). Therefore, we conclude that the stability of this RNA stem-loop is directly related to *IGF1R* translation and our mutation of interest works through stabilizing RNA structure. Although it is still unknown how exactly this RNA structure change leads to increased translation, this provides an example of how mutations in low secondary structure areas without obvious sequence motifs can be functional. These CRISPR-based studies demonstrate that single-nucleotide patient 3′ UTR mutations can have significant effects in endogenous contexts, both on a molecular and cellular level.

### Functional oncogenic 3′ UTR mutations correlate with poor patient outcomes

We have discovered individual 3′ UTR mutations that increase oncogenic expression and cell growth, leading us to question how such mutations affect patient outcomes. Extensive patient outcome data are available for the UW TAN patients, allowing us to interrogate how 3′ UTR mutations associate with patient prognosis (Table S6A). To understand the nuances of this relationship, we performed a literature search to curate a list of presumed oncogenic mutations: those that increase expression of an established oncogenic mRNA or decrease expression of an established tumor suppressive gene in our MPRAs (Tables S6B and S6C).

**Figure 7.**
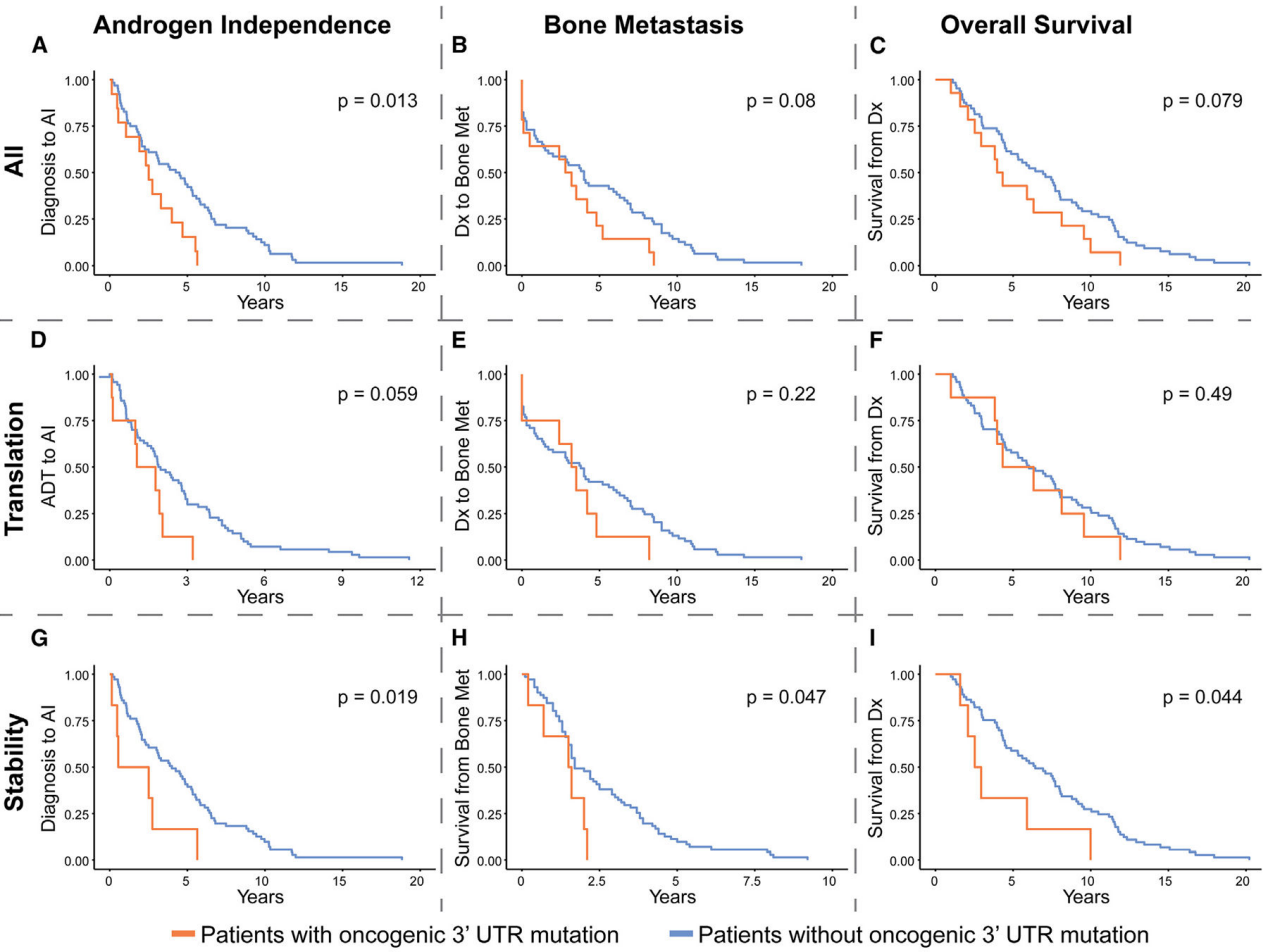
Functional oncogenic 3′ UTR mutations correlate with poor patient outcomes (A–I) Kaplan-Meier plots comparing patients in which any (A–C), translation-related (D–F), or stability-related (G–I) functional oncogenic 3′ UTR mutations were found versus patients without respective 3′ UTR oncogenic mutations. Statistical analysis conducted using Kaplan-Meier estimate. Further analyses available in Table S6D. Number of patients in each grouping: (A–C) with mutation, n = 14; without mutation, n = 65; (D–F) 8/71; (G–I) 6/73.

Overall, we observe that patient tumors harboring any oncogenic 3′ UTR mutations have significantly faster progression to androgen independence (AI) than tumors without these mutations (Figure 7A). Additionally, these patients trend toward shorter time to bone metastasis and overall survival (Figures 7B and 7C). These are important indicators of patient outcome, as progression from androgen sensitivity to castration resistance, as well as the development of distant metastasis, are major causes of patient mortality. Interestingly, patients with translation-based oncogenic 3′ UTR mutations have more moderate phenotypes, displaying a strong trend toward earlier AI but no change in overall survival or metastasis (Figures 7D-7F). However, stability-based oncogenic 3′ UTR mutations have a larger effect on patient outcome, with tumors harboring these mutations causing patients to have significantly poorer prognosis on all three fronts (Figures 7G-7I). Interestingly, although fewer clinical outcome data are available for the SU2C patients, we find similar prognosis trends in this cohort. Those patients containing oncogenic 3′ UTR mutations affecting either translation or stability are found to have higher PSA levels at the time of progression and a shorter median survival time than patients without oncogenic 3′ UTR mutations (Figures S5A and S5B).

Although overall mutational burden can be a confounding factor when analyzing patient outcomes, we did not find that it correlated with changes in prognosis. Neither the SU2C patients harboring any oncogenic 3′ UTR mutations nor the UW patients harboring either translation or stability-altering mutations have significantly different TMB than other patients (Figures S5C-S5E). The total UW patient population harboring oncogenic 3′ UTR mutations has somewhat higher overall TMB (Figure S5F), but as the differences in prognosis among these groups is not correlated with TMB trends, this does not seem to explain patient outcomes as a whole.

Overall, these studies show that functional 3′ UTR mutations in cancer-related genes can significantly add to the burden of disease in prostate cancer patients.

## DISCUSSION

Our study uses a combination of cutting-edge techniques to uncover the landscape of functional mutations in prostate cancer patient 3′ UTRs. Whole-genome and UTR-specific sequencing identifies thousands of somatic 3′ UTR mutations in patients, enriched in oncogenic signaling pathways and *cis*-regulatory motifs. We find hundreds of patient mutations, many affecting oncogenic proteins and pathways, that significantly change either mRNA stability or translation using dual MPRAs. These mutations represent additional insight into how genes such as *IGF1R, MSI2, MBD2*, and *ASCL1* can be aberrantly regulated in cancer, highlighting the importance of expanding the field’s view of cancer genomics to include alterations in UTRs. For example, the knowledge that a patient harbors a translation-increasing 3′ UTR mutation in *IGF1R* may suggest anti-IGF1R agents as an effective route of therapy, even in the absence of CDS mutations or mRNA expression changes.^82^

Interestingly, most functional 3′ UTR mutations alter known regulatory motifs, and in fact the presence of a *cis*-regulatory element at the site of a mutation is found to predict altered function. Additionally, high sequence conservation and low RNA structural stability are associated with functional mutations, agreeing with previous studies that also observed correlations between 3′ UTR function and low RNA structure.^22,83^ These findings contribute to our goal of high-resolution 3′ UTR understanding, where *in silico* analysis of the conservation, sequence, and structure of tumor 3′ UTR mutations may accurately predict mutation function. To fully understand the design principles of 3′ UTRs will likely require testing many more 3′ UTR mutations across different conditions and integrating these results with machine learning.

Specificity and applicability of CRISPR editing has recently increased significantly, allowing deeper understanding of single-nucleotide mutations in endogenous contexts.^84^ We have leveraged this technology to demonstrate that patient 3′ UTR mutations in *ZWILCH* and *IGF1R* have oncogenic cellular effects. Importantly, we observed significant changes in both protein expression and growth even when these mutations were only introduced in a heterozygous manner. As mutations are often sub-clonal and/or heterozygous in tumors, this demonstrates that 3′ UTR mutations can impactfully tune oncogenic expression even when present at low variant allele fractions and cause significant additive effects on cellular oncogenicity. These endogenous mutation models allow accurate determination of molecular mechanisms within mutations’ genomic context, allowing the discovery of RBP- and structure-based mechanisms of the *ZWILCH* and *IGF1R* mutations. Expanding the use of CRISPR editing by designing large-scale 3′ UTR endogenous mutation editing screens, as has recently been accomplished for CDS variants, will be integral in further understanding patient 3′ UTR mutations.^85-88^

Our work represents a comprehensive view of the extent to which disease-relevant 3′ UTR mutations affect mRNA stability, TE, and cancer phenotypes, expanding the boundaries of functional cancer genomics and potentially uncovering therapeutic targets in previously unexplored regulatory regions.

### Limitations of the study

Our MPRAs were limited to the mCRPC model of PC3 cells; however, it is encouraging to note that only 1 of 6 separately tested mutations functioned differently in LNCaP versus PC3 cells and that other UTR-based MPRAs have found high correlation in UTR function across cell types,^17^ suggesting that our results are likely applicable to other cellular contexts. Our study is also limited by the necessity of using 201 base pair fragments of 3′ UTRs in our reporter library, as cloning full-length 3′ UTRs would be impossible when 3′ UTRs are usually thousands of bases long and the maximum next-generation sequencing read length is only ~300 nt. Although this design does not incorporate long-range interactions between distant 3′ UTR, 5′ UTR, or CDS regulatory elements and the mutations of interest, it highlights the effects of altering specific regulatory motifs, which are usually 5–20 bases long. To truly capture such complex interaction networks, mutations must be studied in their endogenous genomic context.

## STAR★METHODS

### RESOURCE AVAILABILITY

#### Lead contact

Further information and requests for resources and reagents should be directed to and will be fulfilled by the lead contact, Dr. Andrew C. Hsieh (ahsieh@fredhutch.org).

#### Materials availability

Plasmids and mutant cell lines generated in this study are available upon request to the lead contact.

#### Data and code availability

- MPRA sequencing data for plasmid library sequencing, polysome profiling MPRA, and IVT MPRA is deposited under superseries GSE200304.
- All original code has been deposited at Zenodo and is publicly available. DOI is listed in the key resources table.
- Any additional information required to reanalyze the data reported in this paper is available from the lead contact upon request.

### EXPERIMENTAL MODELS AND SUBJECT DETAILS

#### Cell culture

Cell lines used in this study are PC3, HEK293T, and LNCaP cells. Each were cultured primarily in RPMI 1640 media + L-glutamine (Gibco) supplemented with 10% fetal bovine serum (Cytiva), 1% penicillin/streptomycin (Gibco), and 1% L-glutamine (Gibco), except where noted for treatment. Cell lines were cultured in an incubator at 37°C with 5% CO_2_.

### METHOD DETAILS

#### MPRA plasmid library construction

A pool of 13,851 250bp 3′ UTR sequences (Table S2) was manufactured by TWIST Bioscience and resuspended to 10 ng/μL in TE buffer. A 5ng portion of the pool was amplified by PCR using Phusion High-Fidelity DNA Polymerase (NEB) following manufacturer protocol for 25 cycles (Table S7, primers #1–2). The 250bp product was purified by agarose gel size selection and extracted using Monarch DNA Gel Extraction Kit (NEB). An additional purification was done using Monarch PCR & DNA Cleanup Kit (NEB). Plasmid backbone pLuc2CP-noARE was linearized by PCR using Q5 High-Fidelity DNA Polymerase (NEB) following manufacturer protocol for 35 cycles, using 5ng input DNA with a 6 min extension time (Table S7, primers #3–4). The 6,895bp product was purified using Monarch PCR & DNA Cleanup Kit (NEB). To remove template plasmid, the linearized vector was digested with DpnI (NEB) in rCutSmart Buffer (NEB) at 37°C for 15 min.

The 3′ UTR insert sequence pool and linearized pLuc2CP-noARE were assembled into plasmids using Gibson Assembly Master Mix (NEB) with a ratio of 1:15 backbone to fragment DNA. The reaction was incubated at 50°C for 60 min. Cloning efficiency was enhanced by incubating the completed Gibson reaction at room temp for 60min on a 0.025μm filter (Millipore) floating in a 10cm Petri dish of UltraPure DNase/RNase-Free Distilled Water (ThermoFisher). The reaction was recovered and immediately cloned into Stellar Electrocompetent Cells (Takara) following manufacturer protocol. Transformed cells were plated on 4 LB-ampicillin 24.5cm assay plates (Fisher) to allow for individual colony growth. An aliquot of transformed cells was plated on 10.0cm LB ampicillin plates in a dilution series for quality control and representation calculation. All plates were incubated overnight at 37°C. The bacteria were removed from the assay plates and stored at −20°C until plasmid isolation. 4 colonies from each assay plate were sequenced to confirm 3′ UTR sequence insertion. The Gibson and cloning process was repeated until insert representation of 259x was achieved. Plasmid DNA was isolated using PureLink HiPure Plasmid Filter Maxiprep Kit (Invitrogen).

#### Polysome profiling

PC3 cells were plated at a density of 3 million cells per 15cm plate, with three 15cm plates pooled to make each biological replicate. Cells were transfected with 16μg MPRA plasmid library using 48μL Fugene HD (Promega) per plate 24 h after plating. Cell media was changed to fresh media 16 h after transfection and collected 24 h after media change. At time of collection, cells were trypsinized and pelleted at 300xg for 5 min, then treated with 100 μg/mL (final concentration) cycloheximide (Sigma) for 10 min on ice. After treatment, cells were pelleted and flash frozen until all biological replicates were collected.

Cell pellets were lysed on ice in 220μL of polysome lysis buffer (10 mM Tris-HCl pH 7.4 (Ambion), 132 mM NaCl (Ambion), 1.4 mM MgCl2 (Ambion), 19 mM DTT (Sigma), 142 μg/mL cycloheximide (Sigma), 0.1% Triton X-100 (Fisher), 0.2% NP-40 (Pierce), 607 U/mL SUPERase-In RNase Inhibitor (Invitrogen) with periodic vortex mixing. Lysates were clarified by centrifugation at 9300xg for 5 min and supernatants were transferred to fresh tubes. Three 220μL lysates were combined for each replicate for a total volume of 660μL per sample. This total lysate was split into three parts: 60μL for plasmid DNA extraction, 150μL for total mRNA isolation, and 450μL for polysome profiling.

For each sample, the 450μL lysate fraction was layered onto a 10%–50% (w/v) linear sucrose gradient (Fisher) containing 2 mM DTT (Sigma) and 100 μg/mL heparin (Sigma). The gradients were centrifuged at 37,000 rpm for 2.5 h at 4°C in a Beckman SW41Ti rotor in Seton 7030 ultracentrifuge tubes. After centrifugation, samples were fractionated using a Biocomp Gradient Station by upward displacement into collection tubes, through a Bio-Rad EM-1 UV monitor (Bio-Rad) for continuous measurement of the absorbance at 254 nm. 820μL of TRIzol Reagent (Invitrogen) were added to each RNA fraction and stored at −80°C.

#### *In vitro* transcription

#### MPRA IVT template preparation

DNA template for MPRA *in vitro* transcription was prepared by first digesting 65μg MPRA plasmid library DNA with FseI restriction enzyme (NEB) in rCutSmart buffer (NEB) for ~90 min. FseI singly cuts the MPRA plasmid backbone directly downstream of the luciferase coding sequence and inserted 3′ UTRs. Digested DNA was run on a 1% agarose gel and size extracted using NucleoSpin Gel and PCR Clean-up Kit (Macherey-Nagel). Phenol:chloroform and isopropanol DNA isolation was used to purify extracted DNA, resulting in ~20μg purified, gel-extracted MPRA template.

##### *MPRA* in vitro *transcription*

MPRA template was *in vitro* transcribed, capped, tailed, and purified using the mMESSAGE mMACHINE T7 ULTRA Transcription Kit (Invitrogen). Eleven reactions in total were carried out, each using 1μg of MPRA template in the manufacturer recommended reaction with the addition of 1μLSUPERase-In RNase Inhibitor (Invitrogen). Reactions were incubated at 37°C for 4 h, then 1μL Turbo DNase (Invitrogen) added and incubated at 37°C for an additional 15 min for removal of DNA template. Poly-A tailing protocol was followed to manufacturer’s instructions with a 45-min incubation at 37°C. RNA recovery was performed using lithium chloride precipitation according to kit protocol. All eleven reaction products (~350μg) were pooled to make the MPRA mRNA library.

##### Exogenous spike-in template preparation

DNA template for exogenous control spike-in RNA was prepared from pDualLuc (a generous gift from Arvind Subramaniam) using a two-step PCR approach to add MPRA-compatible ends and T7 promoter to a portion of the nanoluciferase coding sequence. PCRs were carried out using 50μL reactions in Q5 High Fidelity 2X Master Mix (NEB) using pDualLuc-specific primers (Table S7, primers #5–8) and purified using DNA Clean & Concentrator (Zymo Research).

##### *Exogenous spike-in* in vitro *transcription*

Nanoluciferase exogenous spike-in control RNA was *in vitro* transcribed using MAXIscript T7 Transcription Kit (Invitrogen). Protocol was followed to manufacturer’s specifications using 1μg starting material, 1 h incubation at 37°C, and Turbo DNase (Invitrogen) digestion. RNA was purified using lithium chloride precipitation as described in mMESSAGE mMACHINE T7 ULTRA Transcription Kit (Invitrogen).

##### *In vitro* transcription RNA-seq time-course

PC3 cells were plated in 10cm plates at 1.5 million cells per plate. Cells were transfected the day after plating with 8μg *in vitro* transcribed MPRA mRNA library using 40μL Lipofectamine MessengerMAX (Invitrogen) transfection reagent per plate. Cells were washed and media changed 1 h after mRNA transfection to remove residual RNA in media before collection. One 10cm plate of transfected cells was collected for each of 6 biological replicates at 1, 3, 6, 12, and 24 h after transfection, amounting to 30 total plates/samples. Collection was performed on ice by gently scraping cells into cold PBS and pelleting at 350xg for 5 min at 4°C. Pellets were each resuspended in 1mL pre-spiked TRIzol reagent (Invitrogen). TRIzol was spiked with 0.7ng nanoluciferase spike-in control RNA in 35mL total TRIzol (20pg spike-in RNA per sample) for normalization across samples.

#### MPRA sequencing

##### Polysome profiling cDNA and plasmid DNA prep

Total (input), monosome-associated (fraction 5), low polysome-associated (fractions 8 and 9), and high polysome-associated (fractions 10, 11, and 12) mRNA samples were isolated from TRIzol (Invitrogen) using the Direct-zol RNA Miniprep Plus Kit (Zymo Research) with DNaseI treatment according to manufacturer’s directions. RNA was eluted in 50μL nuclease-free water and, for polysome samples, the multiple fractions were then pooled and concentrated using RNeasy MinElute Cleanup Kit (Qiagen). cDNA was made from 1μg RNA per sample using SuperScript III First-Strand Synthesis System (Invitrogen) and MPRA-specific primer (Table S7, primer #9). Plasmid DNA was isolated from 60μL cell lysate fractions using a modified QIAprep Spin Miniprep Kit (Qiagen) protocol, starting with addition of P2 to lysate and continuing manufacturer’s directions from this step.

##### IVT RNA-seq time course cDNA preparation

RNA was isolated from samples collected in TRIzol (Invitrogen) using a chloroform-isopropanol protocol. 200μL chloroform was added to 1mL TRIzol samples, transferred to a heavy phase-lock gel tube (QuantaBio), and spun in a microcentrifuge at 12,000xg for 15 min at 4°C. The aqueous layer was transferred to a new tube containing 1μL of glycogen (Fisher Scientific) and equal volume isopropanol added. After 10 min of incubation at room temperature, sample was spun at 20,000xg for 20 min at 4°C, then RNA pellet was washed with fresh 75% ethanol before drying and resuspending in 40μL nuclease-free water. Isolated mRNA was DNase treated using Turbo DNase (Invitrogen) in reactions consisting of 11 μL (~15μg) RNA and 1.5μL Turbo DNase according to manufacturer’s protocol. DNase-treated RNA was purified using lithium chloride precipitation as described in mMESSAGE mMACHINE T7 ULTRA Transcription Kit (Invitrogen). 2μg RNA per sample was reverse transcribed into cDNA using SuperScript III First-Strand Synthesis System (Invitrogen) and MPRA-specific primer (Table S7, primer #2).

##### Two-step PCR amplicon sequencing library prep

Library pools for MPRA plasmid library, polysome-based MPRA, and IVT-based MPRAs were assembled separately using the same protocol. Each sample (1 for plasmid library, 24 for polysome MPRA, and 30 for IVT MPRA) was individually amplified using Q5 High Fidelity 2X Master Mix (NEB) in a 50μL reaction with 250ng plasmid DNA (MPRA plasmid library), 9.5μL of cDNA or 250ng of plasmid DNA (Polysome MPRA), or 10μL cDNA (IVT MPRA) and MPRA-specific primers (Table S7, primers #10–11). Thermocycling consisted of: 98°C for 30 s; 10 cycles of 98°C for 10 s, 71°C for 30 s, 72°C for 30 s; and a final extension at 72°C for 5 min. 5μL PCR product was run on an agarose gel to confirm size and remaining 45μL was purified using AMPure XP beads (Beckman Coulter) at a 1.8x ratio. Second round PCR was performed using 20μL round 1 purified PCR product in a 50μL total volume Q5 High Fidelity 2X Master Mix (NEB) reaction and IDT for Illumina DNA/RNA UD Indexes (Illumina) primers (Set C used for polysome MPRA, Set A used for IVT MPRA). Thermocycling consisted of: 98°C for 30 s; 10 cycles (8 cycles for MPRA plasmid library) of 98°C for 10 s, 67°C for 30 s, 72°C for 30 s; and a final extension at 72°C for 5 min. 5μL PCR product was run on an agarose gel to confirm size and remaining 45μL was purified using AMPure XP beads (Beckman Coulter). MPRA plasmid library sample was bead purified at a 1.8x ratio. Polysome MPRA samples were bead purified twice, once at a 1.8x ratio then once at a 0.7x ratio. IVT MPRA samples were bead purified once at a 0.7x ratio.

The concentrations of final purified PCR products were measured using a Qubit Fluorometer (Invitrogen) and size/purity was verified using an Agilent 4200 Tapestation. For the polysome and IVT MPRAs, all samples were pooled for sequencing using 1.869ng per sample (polysome MPRA) or 1ng per sample (IVT MPRA). All sequencing was performed by the Fred Hutchinson Cancer Center genomics core. MPRA plasmid library was sequenced on a MiSeq using the Nano PE150 run configuration. Polysome MPRA was sequenced on a HiSeq 2500 using the Rapid PE150 run configuration. IVT MPRA was sequenced on a NextSeq 2000 using the P3 PE150 run configuration.

##### Dual luciferase assays

###### Construct cloning

For *GABRA4, ARHGEF7, CSRNP3, SLC16A7, PRDM16*, and *LSAMP* mutations, individual luciferase plasmids with target 3′ UTRs were cloned out of the total MPRA plasmid library using inverse PCR followed by plasmid ligation. Briefly, PCRs were performed for each plasmid of interest using the MPRA plasmid DNA library as a template, Q5 High-Fidelity 2X Master Mix (NEB), and opposing 3′ UTR mutation-specific primers that would amplify the entirety of the plasmid beginning with the unique mutation region (Table S7, primers #12–29). After amplification, a mixture of Dpn1 (NEB), T4 PNK (NEB), and T4 DNA Ligase (NEB) was added for linearization of the PCR product and removal of the plasmid library template. For *GPRIN3* and *IGF1R* constructs, 300 nucleotide 3′ UTR fragments were ordered from IDT as eBlocks Gene Fragments, each consisting of the mutation of interest, 200 bases of surrounding 3′ UTR sequence context, and 99 bases of flanking sequence homologous to the pLuc2CP-noARE plasmid surrounding the 3′ UTR insert site. These were cloned into the μLuc2CP-noARE plasmid using Gibson Assembly Master Mix (NEB). All final constructs were transformed into NEB 5-alpha Competent *E. coli* and resultant plasmids confirmed by Sanger sequencing.

###### Protein expression assays

For experiments measuring protein expression, PC3 or LNCaP cells were plated at 8,000 cells (PC3) or 18,000 cells (LNCaP) per well in 96-well plates and transfected 24 h later. PC3 cells were transfected using 0.4μL of Fugene HD (Promega), 100ng pLuc2CP-noARE plasmid containing WT or mutant 3′ UTR sequence, and 1ng of control Renilla plasmid (pRL-UBC) per well. LNCaP cells were transfected using 0.3μL Lipofectamine 3000 (Thermo Fisher), 0.15 μL P3000, 75ng pLuc2CP-noARE plasmid, and 1ng pRL-UBC per well. Each WT or mutant construct was transfected into five technical replicate wells per experiment and repeated in four or more biological replicates. Promega’s Dual Luciferase Assay Reporter System was used to lyse cells in PLB and measure luciferase activity 24 h after transfection according to manufacturer’s instructions. Luminescence was read using a Cytation 5 plate reader (BioTek).

###### Translation assays

For experiments measuring translation, PC3 cells were plated at 60,000 cells per well in 12-well plates and transfected 24 h later using 3.5μL Fugene HD (Promega), 500ng pLuc2CP-noARE plasmid, and 5ng of pRL-UBC per well. Each WT or mutant construct was transfected into three wells per experiment and repeated in 2–4 experimental replicates. Cells were collected 24 h after transfection by trypsinization, where 25% of the cell pellet was used for dual luciferase assays as described above and 75% was collected for RNA isolation and RT-qPCR. Changes in translation were calculated by normalizing the dual luciferase assay result (firefly luciferase:renilla luciferase ratio) to the amount of luciferase mRNA (relative RT-qPCR values) from the same sample.

#### Actinomycin D RT-qPCR

PC3 cells were plated at 35,000 cells per well in 24-well plates and transfected 24 h later using 250ng pLuc2CP-noARE, 2ng pRL-UBC, and 2μL Fugene HD (Promega). Each construct was transfected into two or three replicate wells per time point and the entire experiment was repeated 3 times. After 24 h of transfection, cells were treated with 5μg/μL Actinomycin D (MP Biomedicals), then collected in 250μL TRIzol (Invitrogen) at 0, 8, and 24 h after Actinomycin D treatment. Samples were immediately processed upon collection using the Direct-zol RNA Miniprep Plus Kit (Zymo Research) including on-column DNase treatment. cDNA synthesis was performed on equal volumes of RNA using the iScript gDNA Clear cDNA Synthesis Kit (Bio-Rad), introducing a second round of DNase treatment with this kit. qPCR was performed using SsoAdvanced Universal SYBR Green Supermix (Bio-Rad) with primers amplifying firefly luciferase mRNA (Table S7, primers #30–31).

#### CRISPR base editing

A custom R script was used to filter MPRA results by whether they could be CRISPR edited using available base editing systems. This included filtering for A→G or C→T mutations and searching for nearby PAM sites situated at the proper distance from the mutation of interest. 3′ UTR mutations in *ZWILCH* (chr15:66548998 A→G) and *IGF1R* (chr15:98958058 G→A) were chosen for editing.

To edit the *ZWILCH* 3′ UTR mutation into PC3 cells, we used a system consisting of the NG-ABE8e adenine base editor with NG PAM specificity (a gift from David Liu, Addgene plasmid #138491) and a mutation-specific sgRNA plasmid with sgRNA: 5′-TATTATTGTGTATCTTAAGA-3’. To edit the *IGF1R* mutation into HEK293T cells, we used a system consisting of the evoAPOBEC1-BE4-max cytosine base editor with NGG PAM specificity (a gift from David Liu, Addgene plasmid #122611) and a mutation-specific sgRNA plasmid with sgRNA: 5′-GCCGATGAGGGAGAAAGTTC-3’. sgRNA plasmids were cloned from the pFYF1548 EMX1 sgRNA plasmid backbone (a gift from Keith Joung, Addgene plasmid #47508) using the Q5 Site-Directed Mutagenesis Kit (NEB) and sgRNA-specific primers (Table S7, primers #32–35).

To introduce editing systems to cells, 1.25 million PC3 cells (ZWILCH) or 1.75 million HEK293T cells (IGF1R) were plated in a 10cm dish and transfected the next day with 6μg base editor plasmid, 2μg sgRNA plasmid, 200ng pMaxGFP, and 24μL Fugene HD (Promega). For PC3 *ZWILCH* editing, transfected cells were treated with 10nM romidepsin (Selleckchem) to increase editing efficiency^113^ 8 h after transfection, then media was changed to remove reagents 24 h after romidepsin treatment. For 293T *IGF1R* editing, no romidepsin was used, but media was changed 24 h after transfection to remove transfection reagents. Transfected cells were collected by trypsinization 72 h after transfection and resuspended in 500μL PBS for flow cytometry. Cells were sorted based off live/dead and GFP^+/−^ criteria on a Sony SH800 sorter. Live, GFP+ cells were sorted first as single cells directly into 96-well plates for clonal growth, then a second portion of cells was collected in bulk for polyclonal Sanger sequencing. As single-cell colonies grew in 96-well plates, they were transferred to 24-well plates and then 6-well plates before a pellet was collected for monoclonal Sanger sequencing.

Sanger sequencing was completed by isolating genomic DNA from cell pellets using the DNeasy Blood & Tissue Kit (Qiagen), PCR amplifying the region around the 3′ UTR mutations, and Sanger sequencing through Genewiz. PCR amplification was conducted in a 25μL reaction of Phusion High Fidelity PCR Master Mix (Thermo Scientific) using locus-specific primers surrounding each mutation (Table S7, primers #36–39).

#### Western blotting

Cell pellets were lysed using RIPA buffer (Fisher Scientific) supplemented with protease and phosphatase inhibitors (Sigma). 20-30μg protein per sample was run on a polyacrylamide gel and transferred to a PVDF membrane. Proteins were detected using respective primary and HRP-conjugated secondary antibodies, including rabbit anti-IGF1R (Cell Signaling 3027 1:500), rabbit anti-ZWILCH (Abcam 202898 1:1,000), mouse anti-NCL (Abcam 136649 1:1,000), rabbit anti-AUF1 (Cell Signaling 12382 1:1,000), rabbit anti-ELAVL1 (Cell Signaling 12582), rabbit anti-KHSRP (Cell Signaling 5398 1:500), rabbit anti-TIAL1 (Cell Signaling 5137 1:1,000), mouse anti-β-actin (Sigma A5316 1:1,000), goat anti-rabbit (Invitrogen 31460 1:5,000), and goat anti-mouse (Invitrogen 31430 1:5,000).

#### RT-qPCR

For normalization of luciferase assay results to measure translation, total RNA was extracted from cell pellets collected from luciferase-transfected PC3 cells using the RNeasy Plus Mini kit (Qiagen). iScript Reverse Transcription Supermix (Bio-Rad) was used to reverse transcribe cDNA from equal volumes of RNA across samples. qPCR was performed using SsoAdvanced Universal SYBR Green Supermix (Bio-Rad) for firefly luciferase mRNA (Table S7, primers #30–31).

For CRISPR cell lines, total RNA was extracted from cell pellets collected from CRISPR WT and mutant cell lines at two separate times using TRIzol reagent (Invitrogen). Briefly, TRIzol and chloroform were added to lyse cell pellets, and isopropanol used to precipitate RNA from the resultant aqueous layer. iScript Reverse Transcription Supermix (Bio-Rad) was used to reverse transcribe cDNA from 1μg RNA per sample. qPCR was performed using SsoAdvanced Universal SYBR Green Supermix (Bio-Rad) for *ZWILCH, IGF1R*, and *β-actin* (Table S7, primers #40–45).

#### Cell growth assays

4,000 cells per well were plated in 96-well plates using the media (10%FBS, 1%FBS, 0.1%FBS, or 1%CSS [Gemini Bioproducts]) specific to the experimental condition. For cisplatin-treated conditions, media was changed 24 h after plating to include 15μM cisplatin (Sigma Aldrich). Each cell line was plated in five technical replicate wells per condition and three or more biological replicate experiments were performed. Cells were moved 24 h after plating (or immediately following cisplatin treatment) to an IncuCyte S3 or IncuCyte Zoom (Sartorius) for continuous imaging and confluence analysis. Cells were imaged over the course of 72 h and IncuCyte software used to measure change in confluence over this time. For comparison between experimental replicates, all values within an experiment were normalized around a single wildtype cell line.

#### RBP knockdowns

CRISPR clonal PC3 cells (5 ZWILCH WT lines and 4 ZWILCH 3′Mutant lines) were plated at 120,000 cells per well in 6-well plates. The next day, each cell line was transfected in pairs with either a non-targeting control siRNA or a pool of siRNAs targeting AUF1, ELAVL1, NCL, KHSRP, and TIAL1 (Dharmacon) using 9μL Lipofectamine RNAiMAX (Invitrogen) and 50nM total siRNA (10nM each siRNA when pooling). Cells were collected by cell scraping into cold PBS three days after siRNA transfection and pelleted for Western blot analysis.

### QUANTIFICATION AND STATISTICAL ANALYSIS

#### Obtaining publicly available datasets

BAM files for 101 SU2C project tumor/matched normal mCRPC patients were obtained from Quigley et al. (dbGaP accession code phs001648.v2.p1).^3^ BEDtools^94^ “bamtofastq” was used to extract raw sequencing data from BAM files. Fastq files for UW TAN and PDX UTR sequencing data, UW TAN exome sequencing data, and UW PDX ribosome profiling data were downloaded from Lim et al. UW UTR sequencing data (dbGaP: phs001825.v1.p1; UW exome sequencing data (GEO: GSE147250 and GEO: GSE171729); and PDX ribosome profiling data (GEO: GSE130465).^17^

All downloaded fastq files were aligned to hg38 using Bowtie2 (v2.4.2)^95^. The reference used for alignment was downloaded from GDC portal (https://gdc.cancer.gov/about-data/gdc-data-processing/gdc-reference-files/GRCh38.d1.vd1.fa.tar.gz). The reads with low quality were filtered and duplicates were marked using Picard (v2.25.0, http://broadinstitute.github.io/picard) and GATK^96^ (v4.2.5). Coverage for the whole UTR and CDS space as well as for particular 3′ UTRs were calculated using GATK DepthOfCoverage.

#### Subtraction of mouse genome reads

For genome and ribosome profiling sequencing of UW PDX tissues, mouse genome subtraction was used to separate mouse contamination from human tissue sequencing. Short reads were aligned to both human reference genome hg38 and mouse reference genome mm10 separately using STAR2. XenofiltR^97^ was used to retain those alignment records which were found with much higher fidelity in the human genome compared to mouse genome.

#### Somatic mutation analysis

MuTect (v2),^26^ Strelka (v2.9.2)^27^, MuSE(v2.0)^28^, and VarScan2^29^ were used to identify somatic single-nucleotide variants within the UTRs and CDS for each tumor and matched normal pair. For Mutect2, separate panels of normal were constructed for the SU2C and UW datasets using the matched normal samples. Three different bed files were used in separate runs for obtaining 3′UTR, 5′UTR, and CDS mutations: ncbi_hg38_knownGene_utr3_bed.bed, ncbi_hg38_knownGene_utr5_bed.bed, and ncbi_hg38_knownGene_cds_bed.bed, respectively. Annovar^98^ was used for annotating the variants. For SU2C samples obtained from Quigley et al.,^3^ the following cutoffs were applied to derive a final list of mutations: Ref_reads_in_normal>=8, Total_reads_in_Tumor>=28, Alt_reads_in_Tumor>=10, Tumor_VAF>=0.1, Normal_VAF<=0.05. For UW TAN and PDX samples obtained from Lim et al.,^17^ the following cutoffs were applied: Ref_reads_in_normal>=8, Total_reads_in_Tumor>=14, Alt_reads_in_Tumor>=5, Tumor_VAF>=0.1, Normal_VAF<=0.05. Variant allele frequency (VAF) refers to the fraction of sequencing reads overlapping a genomic coordinate that supports the non-reference (mutant/alternate) allele. Mutations obtained from 2 or more callers were retained for all further mutation analysis. Mutations in hypermutant samples 10–068 (UW TAN), DTB-083 (SU2C), and LuCaP147 (UW PDX) were removed from mutational analysis.

#### Tumor mutational burden

Tumor mutational burden (mutations per megabase) for each patient was calculated from all variants (passing filters and called by two or more tools) separately in the 3′ UTR, 5′ UTR and CDS regions as defined by the UCSC knownGene Table.

#### Mutational signature analysis

Mutational signatures for 3′ UTR mutations and CDS mutations were evaluated separately using DeconstructSigs.^99^ The deconvoluted mutation signature frequency was derived using 30 predefined COSMIC SBS V2 signatures.

#### Clonality

Clonality analysis was performed using PyClone^111^ (version 0.13.1) with a beta-binomial model for 10,000 iterations, a burn-in of 1000 and other default parameters were used. For each tumor sample, the input consisted of tumor purity and reference and variant read counts and allele-specific copy number for all filtered, finalized coding region and UTR mutations. Tumor purity, tumor ploidy, and absolute copy number analysis was calculated using Sequenza^112^ (v 3.0.0). A normalization step using hg38 GC content data from the UCSC genome browser was performed to avoid GC-related bias. Sequenza copy number estimation uses the average depth ratio (tumor vs. normal) and B allele frequency (the lesser of the two allelic fractions as measured at germline heterozygous positions) and performs the calculation considering the derived overall tumor ploidy. Pyclone was then run in single-sample mode for all 182 samples. Pyclone results provide a cellular prevalence (CP) estimate that ranges between 0 and 1 for each mutation, where a higher CP indicates that the mutation is clonal.

#### Mutation significance analysis

The R package FishHook^43^ (v 0.1) was used to identify 3′ UTRs that were significantly mutated over background rates genome-wide. Covariates of GC content (calculated from hg38 genome) and chromatin state (ChromHMM from normal prostate sample BSS01459 of the EpiMap project^114^) were used.

#### 3′ UTR mutational motif analysis

Analysis of 3′ UTR mutations within *cis*-element regulatory regions was performed by examining if the observed mutations in our patient cohort disrupt RNA-binding protein motifs, miRNA seed sequences, the polyadenylation signal AAUAAA, or the m^6^A RRACH motif. Position weight matrices describing RNA binding protein motifs were obtained from three sources: the CisBP-RNA database,^115^ RNA Bind-N-Seq project,^58^ and mCross analysis of ENCODE eCLIP data.^116^ Seed sequences of human miRNAs were obtained from miRBase^117^ and the expression of miRNAs in the PC3 cell lines obtained from ENCODE project ENCSR387TQN. Only the top 100 expressed miRNAs in PC3 cells were used for motif analysis to filter for motif changes that could affect binding of only well-expressed miRNAs. A custom set of Python scripts was written to determine whether the observed counts of 3′ UTR mutations were statistically enriched within these regulatory elements when compared to a random model preserving sequence-specific characteristics such as trinucleotide context. In this analysis, mutations that impacted pre-existing regulatory elements or introduced new elements were both considered. First, each observed mutation was analyzed as to whether it added or removed the sequence of each motif. To generate the background distribution, permutations of all 3′ UTR mutation locations found within our dataset were performed ~10,000 times. The original mutational frequency of all specific transversions, transitions, and trinucleotide context (a total of 288 possible mutations are possible under this scheme—64 possible codons plus 32 additional with no nucleotide in exclusively the first or third position, each with three possible mutations to the middle base) were taken into account. The number of mutations in these permutations that affected each type of motif in each database or specific element was counted. The total number of observed mutations impacting each regulatory element type was compared to the background distribution of the permutation data using a one-sample t test.

#### Design of MPRA plasmid library

The MPRA plasmid library consists of 66 control sequences and 6,892 pairs of WT and Mutant sequences based on patient mutations and their surrounding endogenous 3′UTR sequence. All 13,851 3′ UTR insert sequences are 201 nucleotides long. There are three types of control sequences based on miRNA seed sequences,^117^ the PTRE-Seq 3′ UTR MPRA,^19^ and a 3′ UTR MPRA by Oikonomou et al.^20^ For the patient mutation sequences, transcript ids, genomic coordinates, and transcription stop sites for the 3′UTR of each mutated gene were obtained from UCSC’s Refseq Table using R/Bioconductor package GenomicFeatures.^100^ 3′ UTR sequences were retrieved using R/Bioconductor package “BSgenome.Hsapiens.UCSC.hg38” (https://doi.org/10.18129/B9.bioc.BSgenome.Hsapiens.UCSC.hg38). These were used to extract the 100 bases upstream and 100 bases downstream of each mutation of interest.

#### MPRA sequencing and statistical analysis

##### Alignment and read counts

Fastq files were aligned to a custom reference genome consisting of our MPRA library 3′ UTR sequences using Bowtie2^95^ (v2.4.2) allowing for 0 mismatches. WT and mutant barcodes were segregated to make samples for each sample group. Log_2_CPM values were calculated using edgeR.^101^ The normalized count data (log_2_(cpm+1)) was used for Pearson’s correlation analysis in R and visualized using ggplot2.^102^

##### Polysome MPRA statistics

For polysome MPRA statistical analysis, xtail^103^ was used to identify differentially regulated 3′ UTRs. Translation efficiency was calculated by total polysome (high polysome + low polysome) to total RNA and high polysome to total RNA ratios for each 3′ UTR. RNA expression changes, used for internal control validation, were calculated by total RNA to plasmid DNA ratios. A ratio of ratios was then calculated to compare Mutant TE to WT TE for each 3′ UTR mutation and an FDR<0.10 in this comparison was considered significant.

##### IVT MPRA statistics

For IVT MPRA statistical analysis, all counts within each sample were first normalized using the spike-in control counts of that sample. Then, decay ratios were calculated from 1hr-to-3hr and from 1hr-to-6hr. These decay values were compared between paired WT and Mutant 3′ UTR sequences across the 6 replicates using two-sample t-tests. Additionally, non-linear least-squares regression curves were fitted to each 3′ UTR insert to calculate mRNA half-lives. This was done using the nlstools package^104^ in R using the exponential decay function $y\sim \text{yf}+(y0-\text{yf})\;{}^{*}\;\text{exp}(-\text{alpha}\;{}^{*}t)$ and the starting parameters y0 = 0.5, yf = 0, alpha = 0.1. Half-lives were calculated based on resultant alpha values using the relationship half-life = ln(2)/alpha. Mutations that were considered to have significantly changed mRNA stability passed all the following filters: (A) sum of 1hr read counts ≥24 for both WT and Mutant 3′ UTR inserts, (B) p < 0.05 and ∣log_2_FC∣>0.3 in either 3 h or 6 h decay comparison, and (C) ∣log_2_FC half-life∣>0.2.

#### Ribosome profiling data analysis

STAR (v2.7.3a)^105^ was used to align the downloaded fastq files from Lim et al.^17^ (GEO repository GSE130465) to hg38 and subtraction of mouse sequences was performed using XenofilteR.^97^ Aligned reads were counted for gene associations against the UCSC genes database with HTSeq (0.11.0)^106^. Five UW PDX and five normal prostate tissue samples were each sequenced twice. In each analysis, the two replicates for each UW PDX were considered as the test group and five normal prostate tissue samples as the control group. Xtail^103^ (v1.1.15) was used to find translationally regulated genes individually for each LuCaP. DESeq2^107^ was used to determine changes in RNA expression. 3′ UTR mutation-mediated changes in either translation efficiency or RNA expression were calculated by comparing the gene expression values for the tissue sample in which the mutation was found to the average values across the other four non-mutant tissue samples.

#### GSEA analysis

Enrichr^108^ was used to compute overlaps and enrichment of gene sets within the KEGG_2021_Human and Reactome_2019 databases, and gene sets with FDR<0.05 were considered significant.

#### Survival analysis

Literature search was conducted on the genes containing 3′ UTR mutations that significantly changed translation with a log_2_FC > 0.75 and or changed stability with a log_2_FC > 0.6 (UW analysis) or log_2_FC > 0.5 (SU2C analysis). Mutations in established oncogenic genes that increased mRNA translation or stability and mutations in established tumor suppressive genes that decreased mRNA translation or stability were noted as “oncogenic 3′ UTR mutations” (Tables S6B-S6C). Patients in which these mutations were originally called constituted the test set of patients and comparisons made between this group and the patients not bearing oncogenic 3′ UTR mutations. For UW analysis, the survminer R package was used to plot Kaplan-Meier curves and perform default statistical analysis (Table S6D). For SU2C analysis, GraphPad Prism was used to plot data and perform analyses.

#### Sequence conservation analysis

Sequence conservation scores at each mutation were retrieved using the GenomicScores^109^ package in R. The PHASTCONS 100-way vertebrate alignment score was extracted for the location of each mutation of interest from the “phastCons100-way.UCSC.hg38” database.^118^

#### RNA structural stability analysis

RNA secondary structure for each 201bp 3′ UTR fragment of the MPRA plasmid library was predicted using the ViennaRNA package.^80,81^ The minimum free energy algorithm of Zuker & Stiegler 1981, which yields a single optimal structure, was used and the ΔG of these structures was reported.

#### Filtering CRISPR-able mutations

All mutations that significantly altered either mRNA translation or stability were computationally sorted to determine whether they were valid targets for CRISPR base editing. This was done via custom R script that included filtering out non-transition base changes (retaining C→T, G→A, A→G, and T→C), obtaining the sequence around each mutation, and subsequently determining whether there was a PAM sequence nearby in the proper orientation. This required searching the positive or negative DNA strand depending on the base change, as the mutation must be C→T or A→G on the strand that contains the PAM. Multiple possible PAM sequences were allowed, including NGG, NG, NGAN (VQR), NGCG (VRER), NNGRRT (Sa), and NNNRRT (SaKKH), but NGG and NG sites were prioritized. The distance between the PAM and desired mutation was required to be 13–18 bases, and no other bystander mutations were allowed within this editing window (e.g.,: if the desired mutation was C→T, no other cytosines in the window).

#### Measuring CRISPR editing

Copy number at the ZWILCH locus in PC3 cells and the IGF1R locus in 293T cells were obtained by searching publicly available datasets. PC3 copy number analysis was obtained from the Broad DepMap project (CCLE_ABSOLUTE_combined_20181227 and OmicsCNGene.csv) to conclude that PC3 cells are diploid at the ZWILCH 3′ UTR. 293T copy number analysis was obtained from hek293genome.org, concluding that 293T cells are tetraploid at the IGF1R locus. EditR^110^ was used to analyze percent editing from Sanger sequencing traces of clonal cell lines.

#### Plots and statistical analysis

All analysis was done either in R (v4.2.0) with plots made using ggplot2^102^ or in GraphPad Prism.

## Supplementary Material

1

2

3

4

5

6

7

8

## Figures and Tables

**Table:** KEY RESOURCES TABLE

REAGENT or RESOURCE	SOURCE	IDENTIFIER
Antibodies		
Rabbit anti-ZWILCH	Abcam	202898
Mouse anti-NCL	Abcam	136649
Rabbit anti-IGF1R	Cell Signaling	3027; RRID:AB_2122378
Rabbit anti-AUF1	Cell Signaling	12382; RRID:AB_2616009
Rabbit anti-KHSRP	Cell Signaling	5398; RRID:AB_10694649
Rabbit anti-TIAL1	Cell Signaling	5137; RRID:AB_1904169
Rabbit anti-ELAVL1	Cell Signaling	12582; RRID:AB_2797964
Goat anti-mouse	Invitrogen	31430
Goat anti-rabbit	Invitrogen	31460
Mouse anti-b-Actin	Sigma	A5316; RRID:AB_476743
Bacterial and virus strains		
5-alpha Competent E. coli (High Efficiency)	NEB	C2987I
Stellar^®^ Electrocompetent Cells	Takara	636765
Chemicals, peptides, and recombinant proteins		
AMPure XP for PCR Purification	Beckman Coulter	A63880
HyClone Fetal Bovine Serum	Cytiva	SH30396.03
Charcoal:Dextran Stripped Fetal Bovine Serum	Gemini Bio-Products	100–119
Lipofectamine MessengerMAX	Invitrogen	LMRNA003
Lipofectamine RNAiMAX	Invitrogen	13778030
Lipofectamine 3000	Invitrogen	L3000008
SUPERase-In RNase Inhibitor	Invitrogen	AM2696
TRIzol Reagent	Invitrogen	15596–018
TURBO DNase	Invitrogen	AM2238
Actinomycin D	MP Biomedicals	02104658-CF
DpnI	NEB	R0176
FseI	NEB	R0588S
T4 DNA Ligase (2,000,000 U/mL)	NEB	M0202T
T4 PNK	NEB	M0201S
Fugene HD	Promega	E2311
Phase Lock Gel Tube- Heavy	QuantaBio	10847–802
Romidepsin (FK228, Depsipeptide)	Selleckchem	S3020
Cisplatin	Sigma Aldrich	P4394-25MG
Cycloheximide	Sigma Aldrich	C7698
DTT	Sigma Aldrich	43815
Critical commercial assays		
iScript Reverse Transcription Supermix	Bio-Rad	1708841
iScript gDNA Clear cDNA Synthesis Kit	Bio-Rad	1725035
SsoAdvanced Universal SYBR Green Supermix	Bio-Rad	172–5272
MAXIscript T7 Transcription Kit	Invitrogen	AM1312
mMESSAGE mMACHINE T7 ULTRA Transcription Kit	Invitrogen	AM1345
PureLink^™^ HiPure Plasmid Filter Maxiprep Kit	Invitrogen	K210016
SuperScript III First-Strand Synthesis System	Invitrogen	18080–051
NucleoSpin Gel and PCR Clean-up Kit	Macherey-Nagel	740609.5
Gibson Assembly^®^ Master Mix	NEB	E2611
Monarch^®^ DNA Gel Extraction Kit	NEB	T1020
Monarch^®^ PCR & DNA Cleanup Kit	NEB	T1030
Phusion^®^ High-Fidelity DNA Polymerase	NEB	M0530
Q5 High Fidelity 2X Master Mix	NEB	M0492S
Q5 Site-Directed Mutagenesis Kit	NEB	E0554
Q5 High-Fidelity DNA Polymerase	NEB	M0491
Dual Luciferase Reporter Assay System	Promega	E1980
DNeasy Blood & Tissue Kit	Qiagen	69504
QIAprep Spin Miniprep Kit	Qiagen	27104
RNeasy MinElute Cleanup Kit	Qiagen	74204
RNeasy Plus Mini Kit	Qiagen	74134
Phusion High-Fidelity PCR Master Mix with HF Buffer	Thermo Scientific	F531S
Direct-zol RNA Miniprep Plus Kit	Zymo Research	ZR2070
DNA Clean and Concentrator	Zymo Research	11-302C
Deposited data		
MPRA sequencing data (plasmid, polysome, and IVT)	Gene Expression Omnibus	GEO: GSE200304
All custom code	Zenodo/Github	Zenodo: https://doi.org/10.5281/zenodo.8007705
Experimental models: Cell lines		
HEK293T cells	ATCC	CRL-3216
PC3 cells	ATCC	CRL-1435
LNCaP cells	ATCC	CRL-1740
Oligonucleotides		
IDT for Illumina Nextera UDI, Set A	Illumina	20027213
IDT for Illumina Nextera UDI, Set C	Illumina	20042666
ON-TARGETplus Non-targeting Control Pool	Dharmacon	D-001810-10-05
ON-TARGETplus Human HNRNPD siRNA SMARTpool	Dharmacon	L-004079-00-0005
ON-TARGETplus Human NCL siRNA	Dharmacon	L-003854-00-0005
ON-TARGETplus Human ELAVL1 siRNA	Dharmacon	L-003773-00-0005
ON-TARGETplus Human KHSRP siRNA	Dharmacon	L-009490-00-0005
ON-TARGETplus Human TIAL1 siRNA	Dharmacon	L-011405-00-0005
Individual primers for PCR, etc	IDT	Table S7A
eBlocks Gene Fragments (GPRIN3 & IGF1R)	IDT	Table S7B
Recombinant DNA		
evoAPOBEC1-BE4max cytosine base editor	Thuronyi et al.^89^	Addgene 122611
NG-ABE8e adenine base editor	Richter et al.^90^	Addgene 138491
pFYF1548 EMX1 sgRNA plasmid backbone	Fu et al.^91^	Addgene 47508
pLuc2CP-ARE	Younis et al.^92^	Addgene 62857
pDualLuc	Arvind Subramaniam	N/A
Nanoluciferase exogenous spike-in control	This study	N/A
pIGF1R_sgRNA	This study	N/A
pZWILCH_sgRNA	This study	N/A
pLuc2CP-noARE	This study	N/A
pRL-UBC	This study	N/A
Software and algorithms		
ImageJ	Schneider et al.^93^	https://ImageJ.nih.gov/ij/
GraphPad Prism	GraphPad	www.graphpad.com
IncuCyte Base Analysis Software	Sartorius	N/A
BEDtools	Quinlan & Hall^94^	https://bedtools.readthedocs.io/en/latest/t
Bowtie2 (v2.4.2)	Langmead & Salzberg^95^	https://bowtie-bio.sourceforge.net/bowtie2/index.shtmlt
Picard (v2.25.0)	N/A	http://broadinstitute.github.io/picard
GATK (v4.2.5)	Van der Auwera & O’Connor^96^	https://gatk.broadinstitute.org/hc/en-us
XenofiltR	Kluin et al.^97^	https://github.com/NKI-GCF/XenofilteR
Mutect2 (v2)	Benjamin et al.^26^	https://gatk.broadinstitute.org/hc/en-us/articles/360037593851-Mutect2
Strelka (v2.9.2)	Kim et al.^27^	https://github.com/Illumina/strelka
MuSE (v2.0)	Fan et al.^28^	https://bioinformatics.mdanderson.org/public-software/muse/
VarScan2	Koboldt et al.^29^	https://varscan.sourceforge.net/
ANNOVAR	Wang et al.^98^	https://annovar.openbioinformatics.org/en/latest/
DeconstructSigs	Rosenthal et al.^99^	https://github.com/raerose01/deconstructSigs
GenomicFeatures	Lawrence et al.^100^	https://bioconductor.org/packages/GenomicFeatures
BSgenome.Hsapiens.UCSC.hg38	N/A	https://doi.org/10.18129/B9.bioc.BSgenome.Hsapiens.UCSC.hg38
edgeR	Robinson et al.^101^	https://bioconductor.org/packages/edgeR
ggplot2	Wickham^102^	https://ggplot2.tidyverse.org/
xtail (v1.1.15)	Xiao et al.^103^	https://github.com/xryanglab/xtail
nlstools	Baty et al.^104^	https://github.com/aursiber/nlstools
STAR (v2.7.3a)	Dobin et al.^105^	https://github.com/alexdobin/STAR
HTSeq (0.11.0)	Anders et al.^106^	https://htseq.readthedocs.io/en/master/
DESeq2	Love et al.^107^	https://github.com/mikelove/DESeq2
Enrichr	Chen et al.^108^	https://maayanlab.doud/Enrichr/
survminer	N/A	rpkgs.datanovia.com/survminer/
GenomicScores	Puigdevall and Castelo^109^	https://github.com/rcastelo/GenomicScores
ViennaRNA	Lorenz et al.^80^	https://www.tbi.univie.ac.at/RNA/
EditR	Kluesner et al.^110^	http://baseeditr.com/
Pyclone (v0.13.1)	Roth et al.^111^	https://github.com/Roth-Lab/pyclone
Sequenza (v3.0.0)	Favero et al.^112^	https://bitbucket.org/sequenzatools/sequenza/src/master/
FishHook (v0.1)	Imielinski et al.^43^	https://github.com/mskilab/fishHook/tree/master

## Highlights

- 3′ UTR mutations are enriched in cancer-related genes and regulatory elements
- High-throughput assays reveal 3′ UTR mutations alter mRNA translation and stability
- Endogenous CRISPR-edited 3′ UTR mutations increase cellular stress resistance
- Functional oncogenic 3′ UTR mutations correlate with poor patient prognosis
