# Supplementary material for: Multi-level functional genomics reveals molecular and cellular oncogenicity of patient-based 3′ untranslated region mutations
Source: Cell Rep. Author manuscript; Available in PMC 2023 Sep 29. (PMC10540565; doi:10.1016/j.celrep.2023.112840)
Supplement: 1 [file NIHMS1928233-supplement-1.pdf]

Cell Reports, Volume *42*

Supplemental information

Multi-level functional genomics reveals molecular and cellular oncogenicity of patient-based 3′ untranslated region mutations

Samantha L. Schuster, Sonali Arora, Cynthia L. Wladyka, Pushpa Itagi, Lukas Corey, Dave Young, Bethany L. Stackhouse, Lori Kollath, Qian V. Wu, Eva Corey, Lawrence D. True, Gavin Ha, Patrick J. Paddison, and Andrew C. Hsieh

Supplemental Figure 1

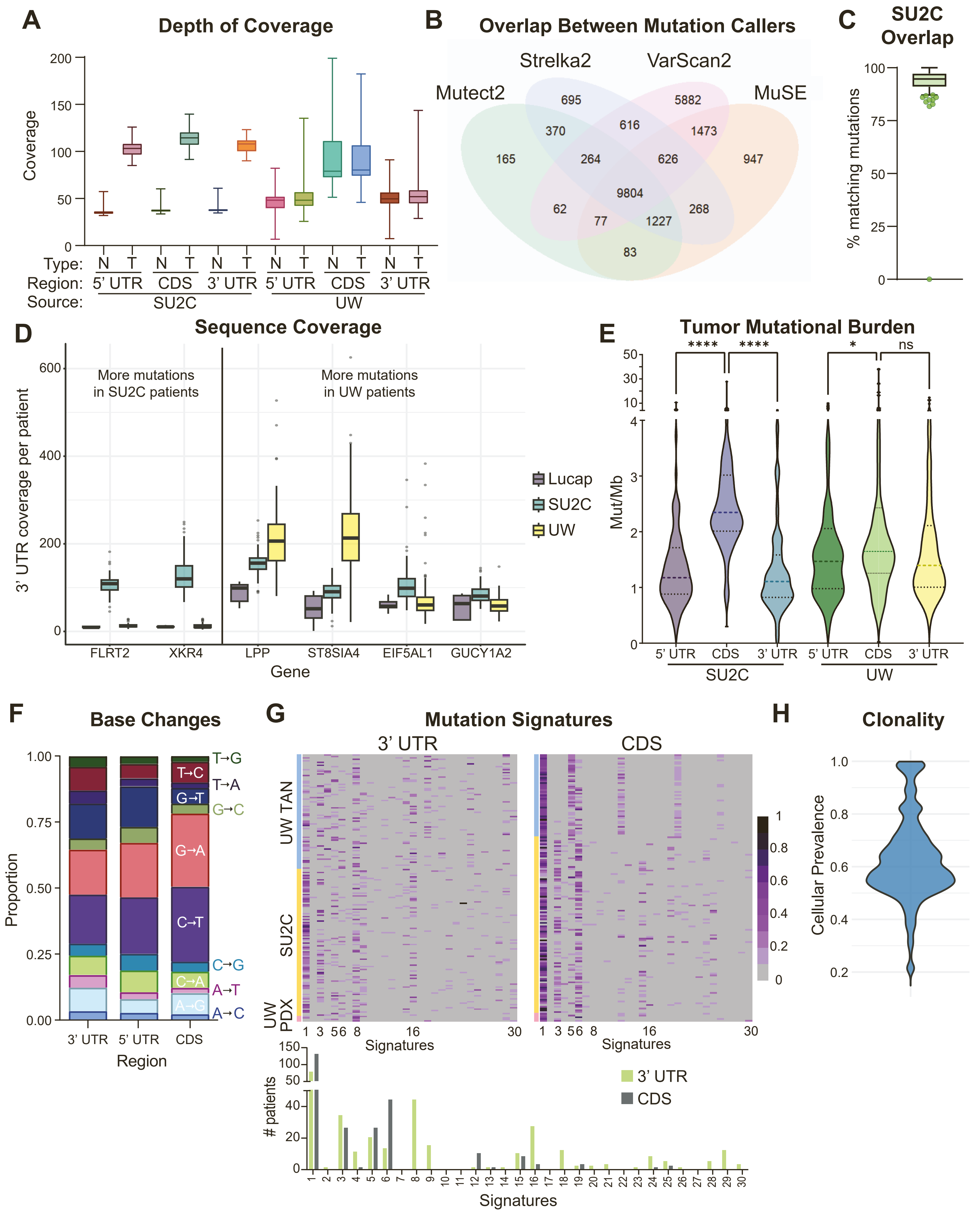

Figure S1: Sequencing statistics and comparison of 3' UTR and CDS mutations; Related to Figure 1

(A) Sequencing coverage of normal (N) and tumor (T) patient samples in each analyzed genomic region. UW group includes both TAN and PDX samples.

(B) Venn diagram showing overlap between 3' UTR mutations called by each mutation caller.

(C) For each SU2C patient, the percent of 3' UTR mutations called by our pipeline that were also called by the original SU2C paper.

(D) Distribution of sequencing coverage across tumor samples for the 3' UTRs in which mutation calling bias was observed between SU2C and UW cohorts. Significant bias was determined by chi-squared analysis, with resultant p-values: *FLRT2* p=0.13; *XKR4, LPP, ST8SIA4, EIF5AL1, GUCY1A2* p<0.05; and all other genes p>0.05. *FLRT2* and *XKR4* are mutated more in the SU2C than UW cohort. *LPP, ST8SIA4, EIF5AL1,* and *GUCY1A2* are mutated more in the UW cohort than SU2C cohort.

(E) Mutations per megabase (Mut/Mb) in each patient by genomic region and patient source. Statistical analysis conducted using two-tailed unpaired t-tests (*p<0.05, ****p<0.0001, ns=p>0.05).

(F) Proportions of each base change called in mutations by genomic region.

(G) Heatmap showing strength of each of 30 COSMIC signatures in 3' UTR (left) and CDS (right) mutations called across patient dataset. Quantified below are the number of patients scoring over 0.2 for each signature.

(H) Cellular prevalence (percent of tumor cells containing a given mutation) for all 3' UTR mutations in our dataset as calculated by the Pyclone R package. Median = 0.60.

Supplemental Figure 2

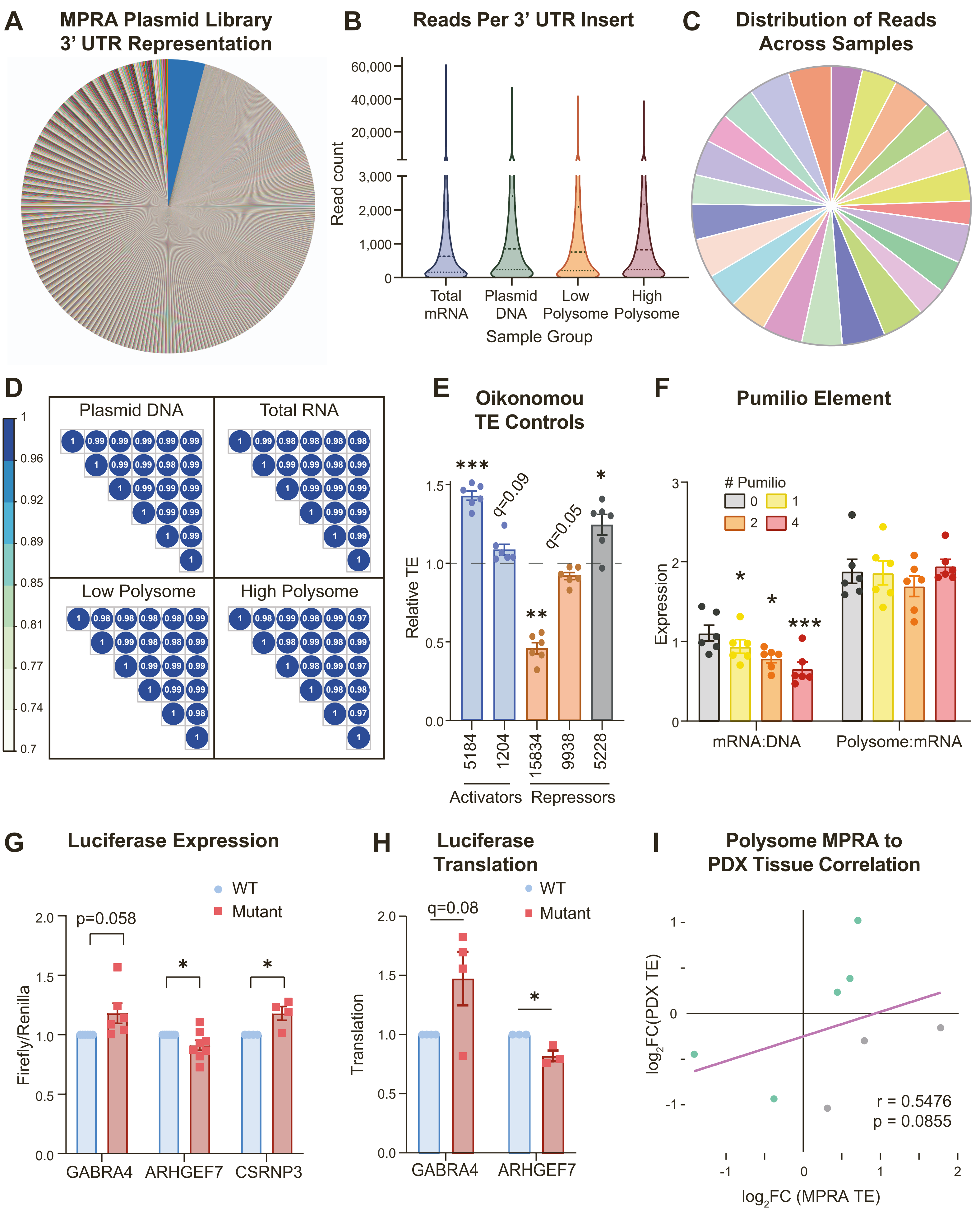

Figure S2: Quality control and validation of MPRA plasmid library and polysome MPRA sequencing; Related to Figure 2

(A) Distribution of 3’ UTR inserts in plasmid library upon DNA sequencing. Blue slice represents blank control vector, amplified erroneously high in this sequencing run only.

(B) Sequencing coverage of each 3’ UTR insert summed across 6 biological replicates upon sequencing of polysome MPRA results, separated by sample type.

(C) Distribution of total sequencing reads in each of 24 samples (6 biological replicates of 4 sample types) sequenced as a pool.

(D) Pearson’s correlation between biological replicates for each sample type.

(E) “Activator” and “repressor” control sequences from Oikonomou *et al.* that were found to significantly change TE (FDR<0.10) in our MPRA results. The control 3’ UTR TE in each biological replicate (n=6) was normalized to the average TE across all 13,851 3’ UTR MPRA inserts for that replicate and this ratio was plotted (mean ± SEM). Colors indicate agreement with expected TE direction. Statistical analysis conducted using ratio paired t-test and multiple comparisons correction of Benjamini, Krieger, and Yekutieli (*q<0.05,**q<0.01, ***q<0.001).

(F) MPRA results for internal control 3’ UTR inserts consisting of a blank vector sequence (0 Pumilio) or a Pumilio element sequence (1, 2, or 4 repeats). Results of these sequences shown for changes in RNA expression (mRNA:plasmid DNA ratio) and TE (total polysome:mRNA ratio) (mean ± SEM, n=6). Statistical analysis conducted using ratio paired t-test and multiple comparisons correction of Benjamini, Krieger, and Yekutieli (*q<0.05, ***q<0.001).

(G) Results of individual dual luciferase assays performed on three functional 3’ UTR mutations. Firefly:Renilla luciferase ratios shown (mean ± SEM), with mutant value normalized to wildtype for each biological replicate (n=6, 8, 4, respectively, with 5 technical replicates each). Statistical analysis conducted using unpaired t-test and multiple comparisons correction of Benjamini, Krieger, and Yekutieli (*q<0.05).

(H) Changes in translation caused by 3' UTR mutations, quantified by dual luciferase assays normalized to luciferase mRNA qPCR ([Firefly RLU/Renilla RLU]/luciferase mRNA, mean ± SEM). Statistical analysis conducted using unpaired t-test and multiple comparisons correction of Benjamini, Krieger, and Yekutieli (*q<0.05), Replicates: n=4 and 3, respectively, with 3 technical replicates each.

(I) Correlation between TE values (total polysome:mRNA ratio) observed for 3' UTR mutations in MPRA (x-axis) and matching TE values obtained from ribosome profiling of UW PDX tissue samples (y-axis). Ribosome profiling expression data was matched by the gene and patient sample in which the 3' UTR mutation was originally found and reported as the change between expression in the mutated tumor sample and the average of the non-mutated samples. Statistical analysis conducted using simple linear regression and Spearman's correlation.

Supplemental Figure 3

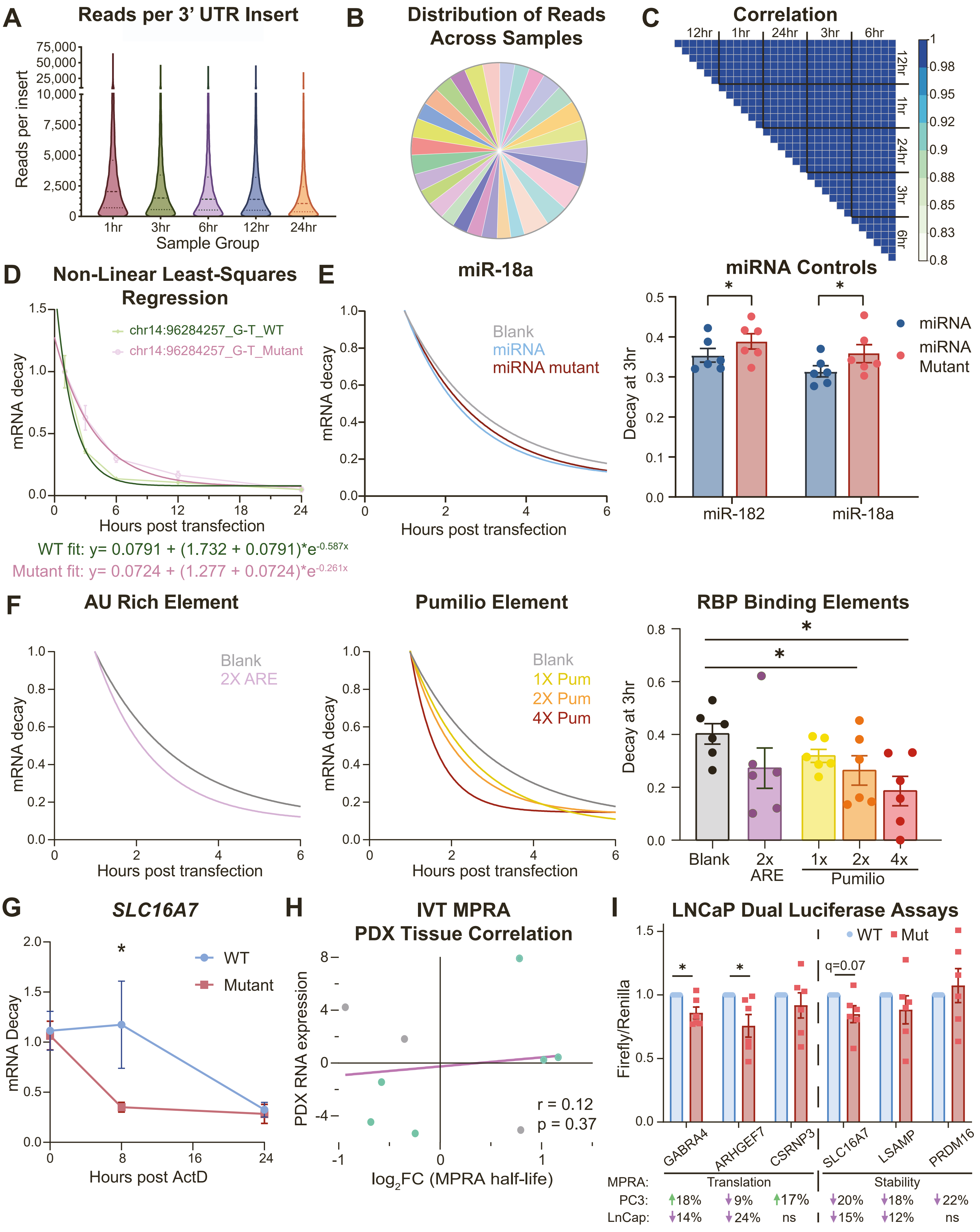

Figure S3: Quality control and validation of IVT MPRA sequencing; Related to Figure 3

(A) Sequencing coverage of each 3' UTR insert summed across 6 biological replicates upon sequencing of MPRA results, separated by time point.

(B) Distribution of total sequencing reads in each of 30 samples (6 biological replicates of 5 sample types) sequenced as a pool.

(C) Pearson's correlation between biological replicates for each sample type.

(D) Example fits of non-linear least-squares regression curves (smooth dark-colored lines) to observed data (straight light-colored lines, mean ± SEM) for a wildtype-mutant 3' UTR pair of inserts. Result fit parameters of each insert shown below.

(E) MPRA results for internal control 3' UTR inserts consisting of a blank vector sequence, miRNA seed sequences, or mutated miRNA seed sequence. NLS curves fit to MPRA data shown for miR-18a (left) and summary results of 1hr to 3hr decay shown for both miR-18a and miR-182 (right, mean ± SEM, n=6). Statistical analysis conducted using ratio paired t-test and multiple comparisons correction of Benjamini, Krieger, and Yekutieli (*q<0.05).

(F) MPRA results for internal control 3' UTR inserts consisting of a blank vector sequence, 2X repeated AU-rich element (ARE), or 1X, 2X, and 4X repeated Pumilio element (Pum). NLS curves fit to MPRA data shown for AU-rich element (left) and Pumilio element (center). Summary results of 1hr-to-3hr decay shown for both (right, mean ± SEM, n=6). Statistical analysis conducted using paired t-test and multiple comparisons correction of Benjamini, Krieger, and Yekutieli (*q<0.05).

(G) Difference in mRNA decay between plasmids containing WT and mutant *SLC16A7* 3' UTR as quantified by qPCR time-course after transcriptional shutoff using actinomycin D (mean ± SEM, n = 7). Statistical analysis conducted using ratio paired t-test at 8hr time-point, *p<0.05.

(H) Correlation between mRNA half-life changes observed for 3' UTR mutations in MPRA (x-axis) and matching RNA expression values obtained from RNA sequencing of UW PDX tissue samples (y-axis). RNA expression was matched by the gene and sample in which the 3' UTR

mutation was originally found and reported as the change between expression in the mutated tumor sample and the average of the non-mutated samples. Statistical analysis conducted using simple linear regression and Pearson's correlation.

(I) Results of individual dual luciferase assays performed in LNCaP cells on six 3' UTR mutations originally found to be functional in PC3 cells. Firefly:Renilla luciferase ratios shown (mean ± SEM), with mutant value normalized to wildtype for each biological replicate (n=6, with 5 technical replicates each). Statistical analysis conducted using unpaired t-test and multiple comparisons correction of Benjamini, Krieger, and Yekutieli (*q<0.05). Summarized below are the differences in mutation effect between PC3 cells (see Figures S2G and 3C) versus LNCaP cells.

Supplemental Figure 4

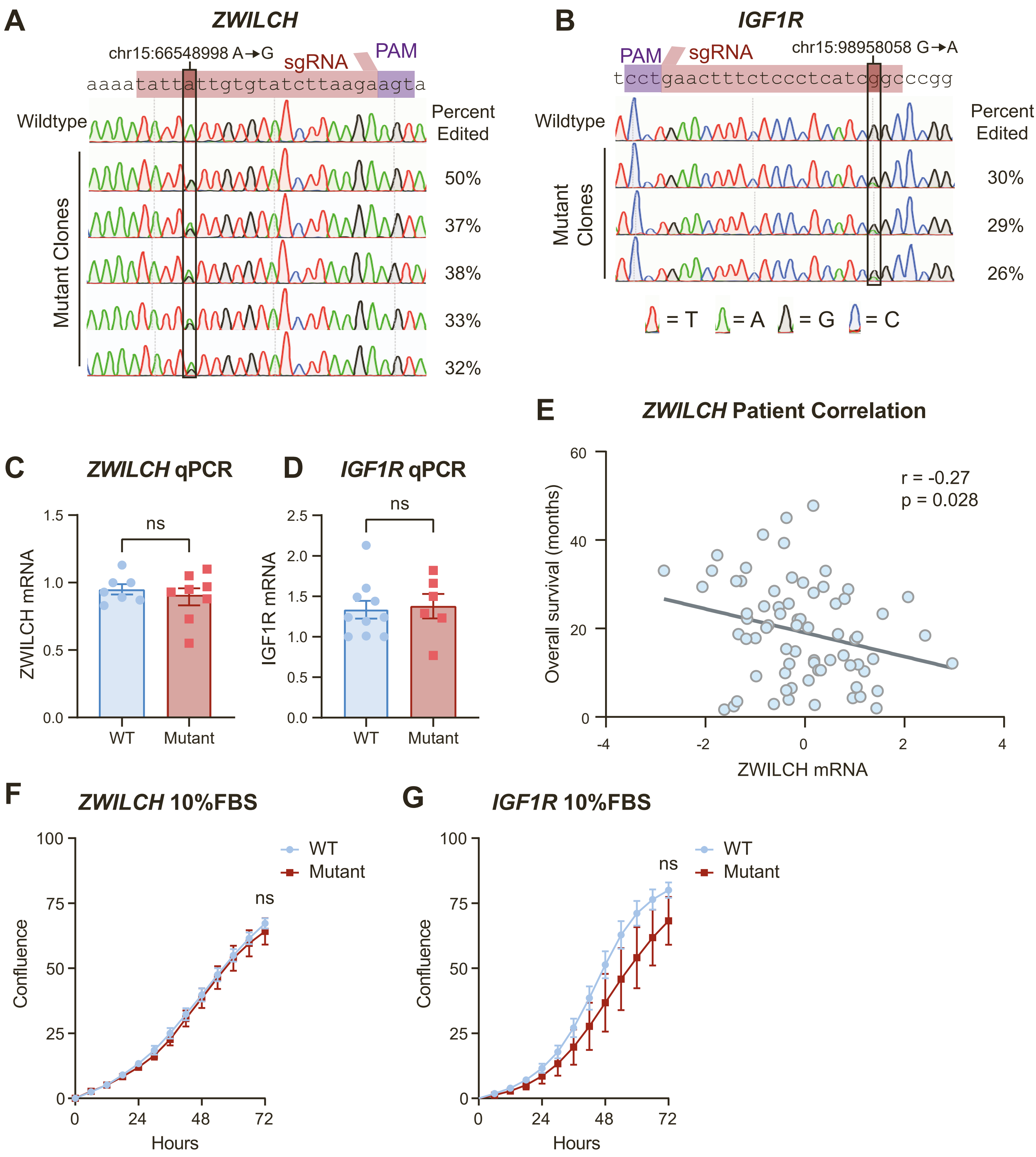

Figure S4: Baseline characterization of CRISPR-edited clonal cell lines and cBioportal mining of *ZWILCH* patient outcome association; Related to Figure 5

(A and B) Sanger sequencing results of *ZWILCH* (A) and *IGF1R* (B) CRISPR-edited clonal cell lines. Percent editing calculated by EditR shown to the right of sequencing traces as estimate of allelic fraction mutated.

(C and D) qPCR results measuring changes in endogenous *ZWILCH* (C) and *IGF1R* (D) mRNA levels relative to housekeeping β-actin in respective wildtype and mutant CRISPR cell lines (mean ± SEM). Statistical analysis conducted using two-tailed unpaired t-tests (ns=p>0.05). Two experimental replicates for each gene shown, with n=7/8 and 10/6 (WT/Mutant lines), for *ZWILCH* and *IGF1R*, respectively.

(E) Patient data obtained from cBioportal (mPCa SU2C/PCF Dream Team 2019 study) with Pearson's correlation between overall patient survival and *ZWILCH* mRNA expression (*ZWILCH*: mRNA expression z-scores relative to all samples, log FPKM capture).

(F and G) Growth kinetics of *ZWILCH* (F) and *IGF1R* (G) wildtype and 3' mutant cell lines under normal conditions (media with 10%FBS), shown as mean ± SEM confluence over time. Result of unpaired t-test of confluence difference at 72 hours is shown (ns=p>0.05). ZWILCH: n=4 experimental replicates and n= 20/19 biological replicates (WT/Mutant lines); IGF1R: n=2 experimental replicates and n= 10/6 biological replicates (WT/Mutant lines); all with n=5 technical replicates each.

Supplemental Figure 5

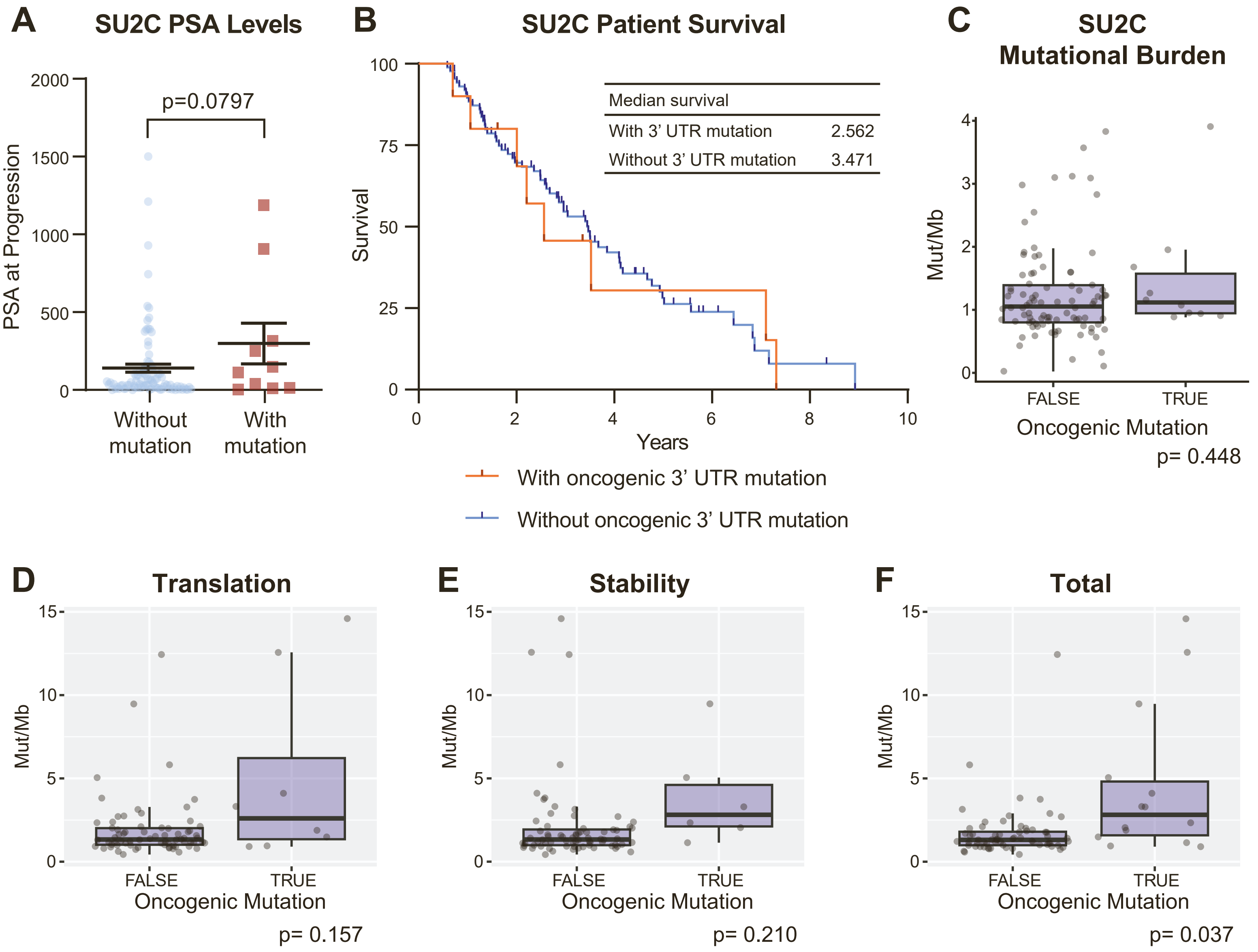

Figure S5: SU2C patient data and tumor mutational burden across patient sets; Related to Figure 7

(A-C) PSA level at progression (mean ± SEM) **(A)**, survival from diagnosis **(B)**, and tumor mutational burden **(C)** for SU2C patients with or without functional oncogenic 3' UTR mutations. Number of patients included in each group: with mutation = 10, without mutation = 91. Statistical analyses conducted using two-tailed unpaired t-tests (A and C).

(D-F) Mutations per megabase (Mut/Mb) in each UW patient in which translation-related **(D)**, stability-related **(E)**, or any **(F)** functional oncogenic 3' UTR mutations were found versus patients without respective 3' UTR oncogenic mutations. Statistical analysis conducted using two-tailed unpaired t-tests.
